# Mobile genetic elements in the genome of the beneficial rhizobacterium *Pseudomonas fluorescens *Pf-5

**DOI:** 10.1186/1471-2180-9-8

**Published:** 2009-01-13

**Authors:** Dmitri V Mavrodi, Joyce E Loper, Ian T Paulsen, Linda S Thomashow

**Affiliations:** 1 Department of Plant Pathology, Washington State University, Pullman, WA 99164-6430, USA; 2 USDA-ARS Horticultural Crops Research Laboratory, 3420 N. W. Orchard Ave, Corvallis, OR 97330, USA; 3 Department of Chemistry and Biomolecular Sciences, Macquarie University, Sydney, NSW, 2109, Australia; 4 USDA-ARS Root Disease and Biocontrol Research Unit, Washington State University, Pullman, WA 99164-6430, USA

## Abstract

**Background:**

*Pseudomonas fluorescens *Pf-5 is a plant-associated bacterium that inhabits the rhizosphere of a wide variety of plant species and and produces secondary metabolites suppressive of fungal and oomycete plant pathogens. The Pf-5 genome is rich in features consistent with its commensal lifestyle, and its sequence has revealed attributes associated with the strain's ability to compete and survive in the dynamic and microbiologically complex rhizosphere habitat. In this study, we analyzed mobile genetic elements of the Pf-5 genome in an effort to identify determinants that might contribute to Pf-5's ability to adapt to changing environmental conditions and/or colonize new ecological niches.

**Results:**

Sequence analyses revealed that the genome of Pf-5 is devoid of transposons and IS elements and that mobile genetic elements (MGEs) are represented by prophages and genomic islands that collectively span over 260 kb. The prophages include an F-pyocin-like prophage 01, a chimeric prophage 03, a lambdoid prophage 06, and decaying prophages 02, 04 and 05 with reduced size and/or complexity. The genomic islands are represented by a 115-kb integrative conjugative element (ICE) PFGI-1, which shares plasmid replication, recombination, and conjugative transfer genes with those from ICEs found in other *Pseudomonas *spp., and PFGI-2, which resembles a portion of pathogenicity islands in the genomes of the plant pathogens *Pseudomonas syringae *and *P. viridiflava*. Almost all of the MGEs in the Pf-5 genome are associated with phage-like integrase genes and are integrated into tRNA genes.

**Conclusion:**

Comparative analyses reveal that MGEs found in Pf-5 are subject to extensive recombination and have evolved in part via exchange of genetic material with other *Pseudomonas *spp. having commensal or pathogenic relationships with plants and animals. Although prophages and genomic islands from Pf-5 exhibit similarity to MGEs found in other *Pseudomonas *spp., they also carry a number of putative niche-specific genes that could affect the survival of *P. fluorescens *Pf-5 in natural habitats. Most notable are a ~35-kb segment of "cargo" genes in genomic island PFGI-1 and bacteriocin genes associated with prophages 1 and 4.

## Background

Recent analyses of bacterial genomes have revealed that these structures are comprised of a mixture of relatively stable core regions and lineage-specific variable regions (also called genomic islands (GIs)), which commonly contain genes acquired via horizontal gene transfer. In bacteria, horizontal gene transfer occurs via conjugation, DNA uptake, transduction and lysogenic conversion, and is mediated largely by mobile genetic elements (MGEs). MGEs are present in most sequenced genomes and can account for the bulk of strain-to-strain genetic variability in certain species [1]. MGEs are part of a so-called "flexible gene pool" and shape bacterial genomes by disrupting host genes, introducing novel genes and triggering various rearrangements. One class of MGEs is derived from bacteriophages and a second is derived from plasmids. Both classes may be associated with integrase genes, insertion sequence (IS) elements and transposons, thus forming elements that are mosaic in nature [2]. Our current knowledge of the impact of MGEs on their hosts comes primarily from pathogenicity islands in which bacteriophages, plasmids and transposons act as carriers of genes encoding toxins, effector proteins, cell wall modification enzymes, fitness factors, and antibiotic and heavy metal resistance determinants in pathogenic bacteria. Much less is known about the diversity and role of MGEs in nonpathogens, in which these elements may enable their hosts to adapt to changing environmental conditions or colonize new ecological niches.

The present study presents the results of an in-depth structural analysis of the MGEs in the genome of *Pseudomonas fluorescens *Pf-5, which is the largest *Pseudomonas *genome sequenced to date http://www.pseudomonas.com[3]. Strain Pf-5 is a model biological control agent that inhabits the rhizosphere of plants and suppresses diseases caused by a wide variety of soilborne pathogens [3-15]. The original analysis of the Pf-5 genome [3] focused primarily on the strain's metabolic capacity and on the pathways involved in the production of secondary metabolites. The latter encompass nearly six percent of the genome and include antibiotics that are toxic to plant pathogenic fungi and Oomycetes and contribute to Pf-5's broad-spectrum biocontrol activity. The aim of the present study was to more thoroughly analyze and annotate sections of the Pf-5 genome that contain MGEs. Here, we describe one transposase, six regions containing prophages (termed Prophage 01 to 06) and two genomic islands that are present in the Pf-5 genome.

## Results and discussion

The genome of *P. fluorescens *Pf-5 contains six prophage regions that vary in G+C content from 62.6% to 46.8% and two putative genomic islands (Table 1). Three of the prophages exceed 15 kb in length and contain genes for transcriptional regulators, DNA metabolism enzymes, structural bacteriophage proteins and lytic enzymes.

**Table 1:** Phage-related elements and genomic islands of *P. fluorescens *Pf-5 genome

Feature	Gene range	5' end	3' end	Size (bp)	%GC	Presence of integrase	Type of feature
Prophage 01	PFL_1210 to PFL_1229	1386082	1402957	16875	62.6	No	SfV-like prophage
Prophage 02	PFL_1842 to PFL_1846	2042157	2050549	8392	46.8	Yes*	Defective prophage in tRNA^Ser^
Prophage 03	PFL_1976 to PFL_2019	2207060	2240619	33559	61.2	Yes	P2-like prophage
Prophage 04	PFL_2119 to PFL_2127	2338296	2351794	13498	56.3	Yes	Defective prophage in tRNA^Pro^
Prophage 05	PFL_3464 to PFL_3456	3979487	3982086	2599	55.3	Yes*	Defective prophage in tRNA^Cys^
Prophage 06	PFL_3739 to PFL_3780	4338335	4395005	56670	57.3	Yes	Lambdoid prophage in tRNA^Ser^
Genomic island 1 (PFGI-1)	PFL_4658 to PFL_4753	5378468	5493586	115118	56.4	Yes	Putative mobile island PFGI-1 in tRNA^Lys^
Genomic island 2 (PFGI-2)	PFL_4977 to PFL_4984	5728474	5745256	16782	51.5	Yes	Genomic island in tRNA^Leu^

*, the predicted integrase gene contains frameshift mutation(s).

### Prophage 01 of Pf-5 and homologous prophages in closely related strains

**Figure 1:**
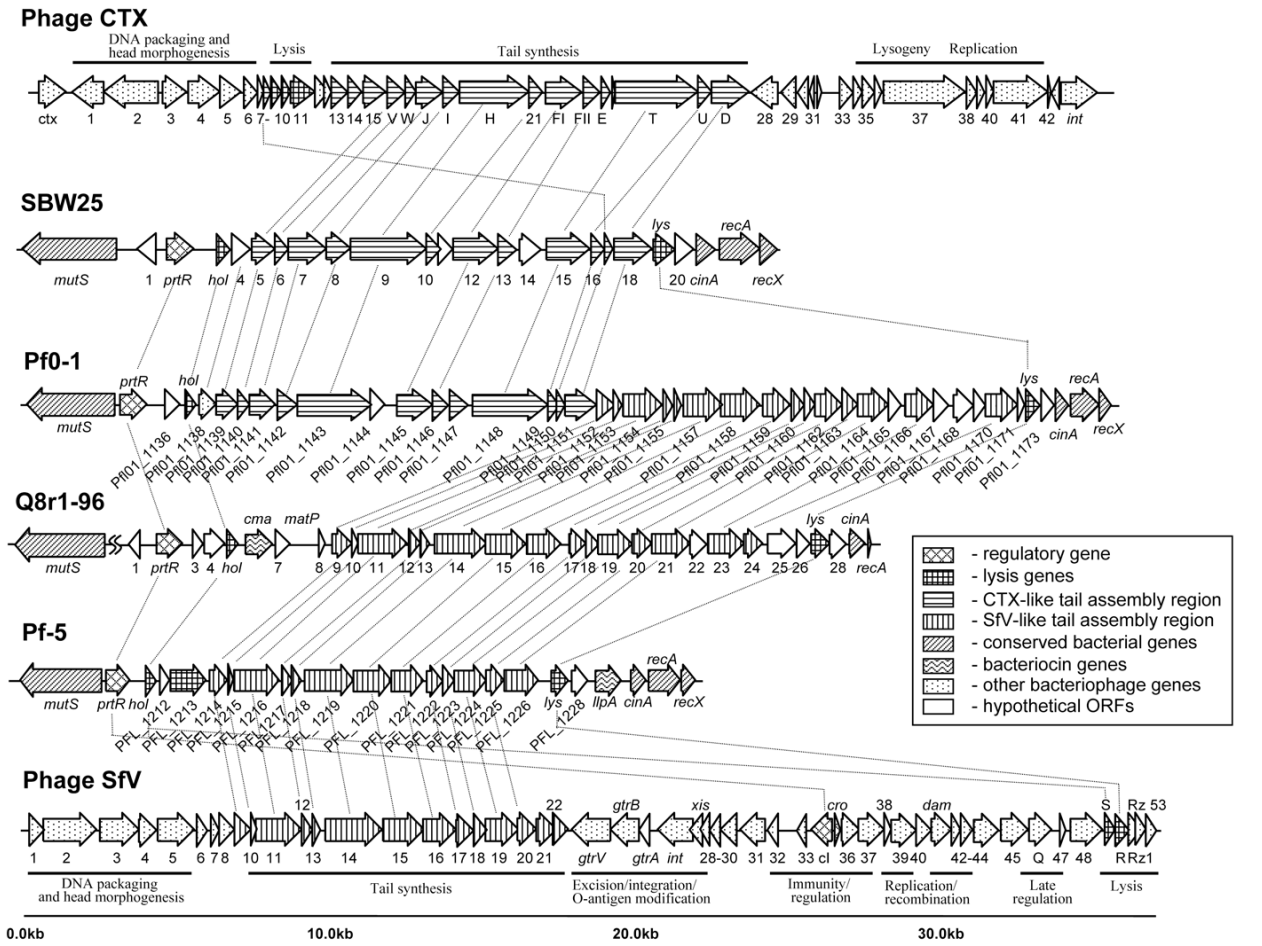
**Organization of prophage 01 from *P. fluorescens *Pf-5 **[49], **related prophages in the *mutS-recA *region of the genomes of other *P. fluorescens *strains, and bacteriophages CTX **[81]**and SfV **[16]. Predicted open reading frames and their orientation are shown by arrows shaded according to their functional category. Homologous ORFs are connected with lines.

Prophage 01 spans 16,875 bp and consists of genes encoding a myovirus-like tail, holin and lysozyme lytic genes, a putative chitinase gene (PFL_1213), and genes for a repressor protein (PFL_1210) and a leptin binding protein-like bacteriocin, LlpA1 (PFL_1229) (Fig. 1, see Additional file 1). The tail assembly gene cluster encodes tail sheath (PFL_1216), DNA circulation (PFL_1216), baseplate assembly (PFL_1222), and tail fiber (PFL_1226) proteins that closely resemble their counterparts from the long contractile tail of the serotype-converting bacteriophage SfV of *Shigella flexneri *[16].

**Figure 2:**
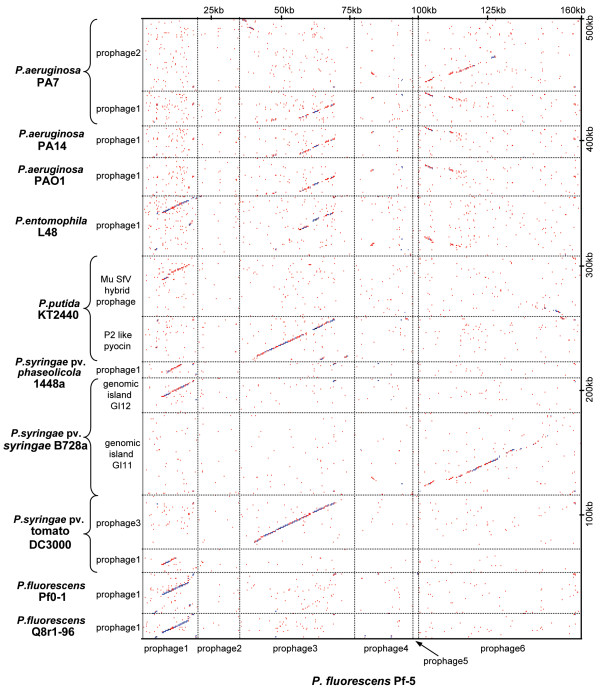
**Dot plot comparison of *P. fluorescens *Pf-5 prophages with similar prophage regions in the genomes of *P. fluorescens *Q8r1-96 **[GenBank EU982300], ***P. fluorescens *Pf0-1 **[GenBank CP000094], ***P. syringae *pv. *tomato *DC3000 **[24], ***P. syringae *pv. *syringae *B728a **[36], ***P. syringae *pv. *phaseolicola *1448a **[37], ***P. putida *KT2440 **[25], ***P. aeruginosa *PA01 **[82], ***P. aeruginosa *UCBPP-PA14 **[35], **and *P. aeruginosa *PA7 **[GenBank CP000744]. All prophage sequences were extracted from genomes, concatenated and aligned using a dot plot function from OMIGA 2.0 with a sliding window of 45 and a hash value of 6. Genome regions used in the analysis encompass open reading frames with following locus tags: *P. fluorescens *Pf0-1 prophage1 – Pfl01_1135 through Pfl01_1173; *P. syringae *pv. *tomato *DC3000 prophage1 – PSPTO_0569 through PSPTO_0587; *P. syringae *pv. *tomato *DC3000 prophage3 – PSPTO_3385 through PSPTO_3432; *P. syringae *pv. *syringae *728a genomic island GI11 – Psyr_2763 through Psyr_2846; *P. syringae *pv. *syringae *728a genomic island GI12 – Psyr_4582 through Psyr_4608; *P. syringae *pv. *phaseolicola *1448a prophage1 – PSPPH_0650 through PSPPH_0671; *P. putida *KT2440 P2 like pyocin – PP3031 through PP3066; *P. putida *KT2440 Mu SfV hybrid prophage – PP3849 through PP3920; *P. entomophila *L48 prophage1 – PSEEN4129 through PSEEN4186; *P. aeruginosa *PAO1 prophage1 – PA0610 through PA0648; *P. aeruginosa *PA14 prophage1 – PA14_07950 through PA14_08330; *P. aeruginosa *PA7 prophage1 – PSPA7_0754 through PSPA7_0789; *P. aeruginosa *PA7 prophage2 – PSPA7_2366 through PSPA7_2431.

We (D.V.M. and L.S.T.) previously identified a highly similar prophage element during a study focused on genetic traits contributing to colonization of the plant rhizosphere by *P. fluorescens*. In that project [17], we applied genomic subtractive hybridization to two strains of *P. fluorescens*, Q8r1-96 and Q2-87, which differ in their ability to colonize wheat roots. Among 32 recovered Q8r1-96-specific loci was a clone dubbed *ssh6*, which proved to constitute part of a 22-kb prophage element that closely resembles prophage 01 of strain Pf-5 (Figs. 1 and 2; see Additional file 2). Like its counterpart, the *ssh6 *prophage from Q8r1-96 carries genes for a myovirus-like tail (*orf10 *through *orf21*), the lytic enzymes holin (*hol*) and endolysin (*lys*), and a Cro/CI-like repressor protein (*prtR*) (Fig. 1; see Additional file 2). Genes in the Q8r1-96 cluster that are not present in Pf-5 encode a colicin M-like bacteriocin (*cma*), a tail collar protein (*orf23*), and putative tail fiber proteins (*orf22 *and *orf25*). Interestingly, the colicin M-like ORF from the *ssh6 *prophage of Q8r1-96 also encodes an enzymatically active protein although the range of microorganisms sensitive to this bacteriocin is currently unknown (Dr. Dominique Mengin-Lecreulx, Institut de Biochimie et Biophysique Moléculaire et Cellulaire, Université Paris-Sud, Orsay, France; personal communication).

**Figure 3:**
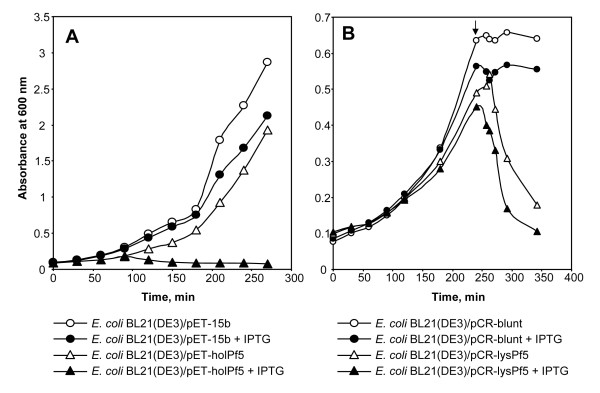
**Lytic activity associated with the prophage 01 of *P. fluorescens *Pf-5**. Putative holin (PFL_1211) (A) and endolysin (PFL_1227) (B) genes encoded by prophage 01 from *P. fluorescens *Pf-5 were cloned in the plasmid vector pCR-Blunt (Invitrogen) under the control of the IPTG-inducible T7 promoter. Broth cultures of *E. coli *Rosetta/pLysS bearing the cloned holin and endolysin genes were induced with 3 mM IPTG and incubated with shaking for 5 hours while monitoring cell growth by measuring OD_600_. The arrow indicates the time of addition of chloroform to the endolysin-expressing culture. Two independent repetitions of the assay were carried out for each gene and yielded identical results (data not shown).

The homologous prophage elements from Pf-5 and Q8r1-96 have simple overall organization, lack integrase and head morphogenesis genes, and carry conserved regulatory, lytic and lambda-like tail morphogenesis genes also found in phage SfV of *Shigella flexneri *(Fig. 1). Taken together, the results of sequence analyses suggest that these regions are not simple prophage remnants but rather, are similar to F-type pyocins. F-type pyocins were first discovered in *P. aeruginosa *and represent a class of high molecular weight protease- and nuclease-resistant bacteriocins that resemble flexible and non-contractile tails of bacteriophages [18,19]. This notion is further supported by the fact that the putative lytic genes found within Pf-5 prophage 01 (Fig. 3) and Q8r1-96 (data not shown) seem to be fully functional. In non-filamentous bacteriophages and bacteriophage tail-like bacteriocins, the lytic activity is provided by the combined action of the small membrane protein holin and a cytoplamic muralytic enzyme, endolysin [19,20]. During phage-mediated cell lysis, holin permeabilizes the cytoplasmic membrane and allows endolysin, which lacks a secretory signal sequence, to gain access to peptidoglycan. To confirm that the prophage 01-like loci indeed encode functional holin and endolysin, we cloned genes PFL_1211 and PFL_1227 from Pf-5 and their counterparts from Q8r1-96 (Fig. 1) in *Escherichia coli *under the control of an inducible T7 promotor. As shown in Fig. 3, induction of both of the putative holin and endolysin genes by IPTG had a strong lethal effect on the host, resulting in rapid cell lysis. In accordance with the current model of action of holin and endolysin, the lethal effect of the endolysin encoded by PFL_1227 was not apparent unless the cytoplasmic membrane was destabilized by addition of small amount of chloroform to the induced *E. coli *culture (Fig. 3B). Gene induction experiments carried out with putative holin and endolysin genes from the *ssh6 *locus of Q8r1-96 had a similar lytic effect on *E. coli *(data not shown).

Interestingly, a prophage element found in the identical spot (between *mutS *and *cinA*) in the genome of *P. fluorescens *SBW25 http://www.sanger.ac.uk/Projects/P_fluorescens has a similar overall organization but contains a P2-like bacteriophage tail cluster (*orf5 *through *orf18*) similar to that in phage CTX (Fig. 1), thus resembling another class of phage tail-like bacteriocins, the R-type pyocins of *P. aeruginosa *[19]. Furthermore, a homologous region from *P. fluorescens *Pf0-1 (CP000094) contains both the lambda-like and P2-like tail clusters (Fig. 1), making it similar to the hybrid R2/F2 pyocin locus from *P. aeruginosa *PAO1 [19]. The differences in organization of the putative phage tail-like pyocins among these prophages clearly indicate that the corresponding loci are subject to extensive recombination, with a possible recombination hotspot between two highly conserved DNA segments comprised of the phage repressor (*prtR*) and holin (*hol*) genes, and the endolysin (*lys*) gene (Fig. 1).

In strains Pf-5 and Q8r1-96, the putative prophage 01-like pyocins are integrated between *mutS *and the *cinA-recA-recX *genes (Fig. 1), which suggests that these elements might be activated during the SOS response, as is the putative prophage gene cluster integrated into the *mutS/cinA *region of *P. fluorescens *DC206 [21]. The *mutS/cinA *region is syntenic in several Gram-negative bacteria [22], and comparisons reveal that prophage 01-like elements occupy the same site in the genomes of *P. fluorescens *Pf0-1, *P. fluorescens *SBW25, and *P. entomophila *L48 [23], whereas unrelated prophages reside upstream of *cinA *in *P. putida *F1 (GenBank CP000712) and *P. syringae *pv. *tomato *DC3000 [24]. The latter strain, as well as *P. putida *KT2440 [25], carry SfV-like bacteriophage tail assembly clusters elsewhere in the genome.

**Figure 4:**
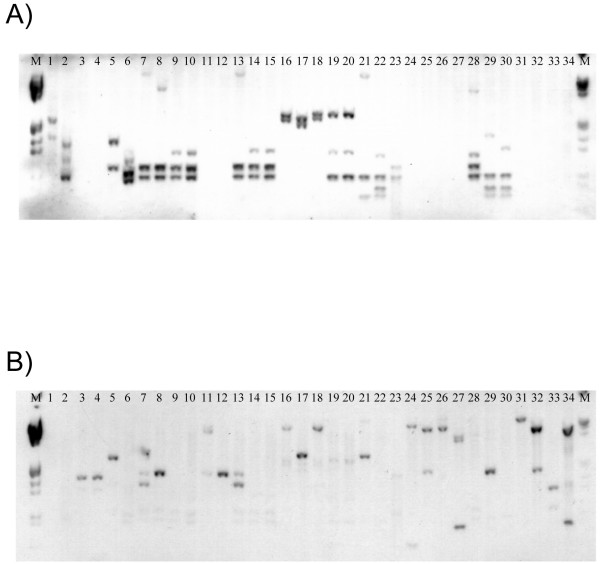
**Southern hybridization of DNA from 34 strains of *P. fluorescens *with probes targeting F-pyocin- and R-pyocin-like bacteriophage tail assembly genes**. Total genomic DNA from each strain was digested with *Eco*RI and *Pst*I restriction endonucleases, separated by electrophoresis in a 0.8% agarose gel, and transferred onto a BrightStar-Plus nylon membrane. The blots were hybridized with biotin-labeled probes prepared from *P. fluorescens *strains Q8r1-96 (A) and SBW25 (B) targeting the SfV-like (A) and CTX-like (B) bacteriophage tail assembly genes, respectively. Strains screened in the experiment are: *P. fluorescens *CHA0 [83], 1; *P. fluorescens *Pf-5 [5], 2; *P. fluorescens *Q2-87 [84], 3; *P. fluorescens *Q2-1 [84], 4; *P. fluorescens *STAD384 [85], 5; *P. fluorescens *Q8r1-96 [74], 6; *P. fluorescens *MVW1-1 [86], 7; *P. fluorescens *FTAD1R34 [85], 8; *P. fluorescens *ATCC49054 [87], 9; *P. fluorescens *Q128-87 [85], 10; *P. fluorescens *OC4-1 [85], 11; *P. fluorescens *FFL1R9 [85], 12; *P. fluorescens *Q2-5 [84], 13; *P. fluorescens *QT1-5 [84], 14; *P. fluorescens *W2-6 [84], 15; *P. fluorescens *Q2-2 [84], 16; *P. fluorescens *Q37-87 [84], 17; *P. fluorescens *QT1-6 [84], 18; *P. fluorescens *JMP6 [84], 19; *P. fluorescens *JMP7 [84], 20; *P. fluorescens *FFL1R18 [84], 21; *P. fluorescens *CV1-1 [84], 22; *P. fluorescens *FTAD1R36 [84], 23; *P. fluorescens *FFL1R22 [84], 24; *P. fluorescens *F113 [88], 25; *P. fluorescens *W4-4 [84], 26; *P. fluorescens *D27B1 [84], 27; *P. fluorescens *HT5-1 [84], 28; *P. fluorescens *7MA12 [86], 29; *P. fluorescens *MVP1-4 [86], 30; *P. fluorescens *MVW1-1 [86], 31; *P. fluorescens *MVW4-2 [86], 32; *P. fluorescens *ATCC17400 [89], 33; *P. fluorescens *SBW25 [90], 34.

The putative F- and R-pyocins appear to be ubiquitously distributed among strains of *P. fluorescens *as illustrated by a screening experiment (Fig. 4) in which genomic DNA of different biocontrol strains was hybridized to probes targeting the lambda-like and P2-like bacteriophage tail gene clusters of Q8r1-96 and SBW25, respectively. The F- and R-pyocin-specific probes each strongly hybridized to DNA from 12 of 34 *P. fluorescens *strains, while the remaining 22 strains tested positive with both probes.

### Prophage 03 of *P. fluorescens *Pf-5

**Figure 5:**
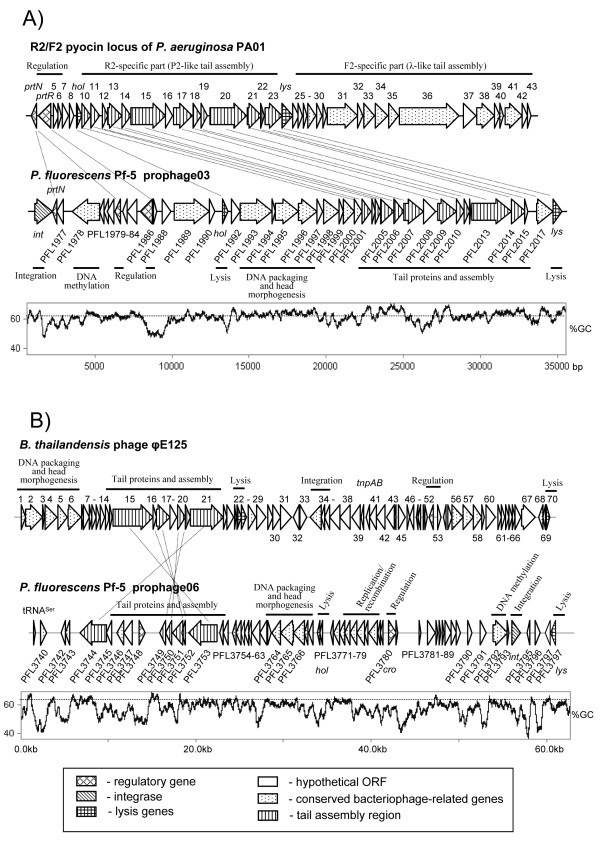
**Comparison of genetic organization of prophages 03 (A) and 06 (B) to that of R2/F2 pyocin locus from *P. aeruginosa *PA01 **[19]**and *B. thailandensis *phage φE125, respectively**. Predicted open reading frames and their orientation are shown by arrows along with a 300-bp sliding window plot of G+C content for the prophage 03 with dotted line tracing the average G+C content (63%) of Pf-5 genome. Predicted ORFs are shaded according to their functional category. Homologous ORFs are connected with lines.

A second large prophage, prophage 03, spans 33.5 kb (Fig. 5A; see Additional file 3) of the Pf-5 genome. Closely related prophages exist in the genomes of *P. putida *KT2440 [25] and *P. syringae *pv. *tomato *DC3000 [24] (Fig. 2) but were not found in *P. fluorescens *strains Pf0-1 or SBW25. Prophage 03 is a chimeric element that contains a siphovirus head morphogenesis region and a myovirus-like tail assembly region (Fig. 5A). The prophage also carries a putative integrase gene (PFL_1976) that encodes an enzyme similar to shufflon recombinases such as the Rci recombinase from plasmid R64 [26], a gene involved in DNA modification (PFL_1978), and a gene for a cytosine-specific methylase (PFL_1979). Genes encoding a LexA-like repressor (PFL_1986), a putative single strand binding protein (PFL_1989), and two genes (PFL_1976 and PFL_1982) with similarity to the pyocin transcriptional activator *prtN *also are present in this region. Holin (PFL_1991) and endolysin (PFL_2018) genes flank a region containing DNA packaging and head morphogenesis and tail assembly genes. The P2-like tail assembly region closely resembles the R2-specific part of R2/F2 pyocin locus of *P. aeruginosa *PA01 [19] (Fig. 5A) and includes genes encoding a tail sheath protein (PFL_2009), a tape measure protein (PFL_2013), a major tail tube protein (PFL_2010), baseplate assembly proteins (PFL_2002 and PFL_2003), and a tail fiber protein (PFL_2007). This region also contains genes involved in head morphogenesis (PFL_1993–1998) that are not present in the R-part of R2/F2-type pyocin cluster of *P. aeruginosa *PA01. Therefore, prophage 03 may represent the genome of a temperate bacteriophage rather than an R-type pyocin.

### Prophage 06 and other prophage regions of *P. fluorescens *Pf-5

Prophage 06 is the largest prophage region of *P. fluorescens *Pf-5 and encodes a 56-kb temperate lambdoid phage integrated into tRNA^Ser^(see Additional file 4). It is mosaic in nature with no homologues present in strains Pf0-1 or SBW25. *P. fluorescens *Pf-5 carries four genomic copies of tRNA^Ser^, of which tRNA^Ser^(2) and tRNA^Ser^(3) are associated with prophages carrying integrases of different specificity (see Additional file 5). The anticodon, V- and T-loops of tRNA^Ser^(2) are parts of the 104-bp putative *attB *site of prophage 06, whereas the T-loop of tRNA^Ser^(3) forms part of the 60-bp putative attachment site of prophage 02. The latter is a prophage remnant that spans 8.4 kb and consists of a gene encoding an ATP-dependent nuclease (PFL_1842) and a phage integrase gene with two internal frameshift mutations (see Additional file 6). The mobility of prophage 06 probably is mediated by a lambda-type integrase encoded by PFL_3794, which resides adjacent to the putative *attR *site. Prophage 06 contains gene modules that are involved in head morphogenesis (capsid proteins PFL_3764 and PFL_3765), DNA packaging (terminase PFL_3766), DNA recombination (a NinG-like protein, PFL_3773 and a putative NHN-endonuclease, Orf1) and tail morphogenesis (tail tip fiber proteins PFL_3744 and PFL_3751, tail length tape measure protein PFL_3753, and minor tail proteins PFL_3749, PFL_3750, and PFL_3752). The tail assembly module resembles the corresponding region from *Burkholderia thailandensis *bacteriophage φE125 [27], although in prophage 06 the module is split by the integration of four extra genes (Fig. 5B). Prophage 06 also contains a regulatory circuit with genes for a Cro/C1 repressor protein (PFL_3780) and two putative antirepressor proteins (PFL_3747 and PFL_3746); a gene for a putative cytosine C5-specific methylase (PFL_3792); and lysis genes encoding holin (PFL_3770) and endolysin (PFL_3798). However, since the endolysin gene is localized beyond the putative *attR *site it is not clear whether it represents part of the prophage 06 genome or a remnant from integration of a different phage (see Additional file 4). Finally, prophage 06 contains two genes, PFL_3740 and PFL_3796, which probably arose through gene duplication and encode putative conserved phage-related proteins that are 88% identical to one another.

Prophages 04 and 05 are prophage remnants with reduced size and/or complexity that carry several mutated phage-related genes (Tables 1, see Additional files 7 and 8). Prophage 04 (13.5-kb) has an average G+C content of 56.3% and contains a putative phage integrase gene adjacent to tRNA^Pro ^(1). It also carries two genes (PFL_2122 and PFL_2123) that encode minor tail assembly proteins, a gene encoding Cro/C1 repressor, and the bacteriocin gene *llpA1 *(PFL_2127). Interestingly, the repressor gene and *llpA1 *are highly similar to their counterparts from prophage 01, suggesting that they arose via gene duplication. Prophage 05, a 2.6-kb prophage remnant, has a G+C content of 55.3% and carries genes encoding a truncated phage integrase and a putative phage tail protein (PFL_3464) (see Additional file 8). The region is flanked by 84-bp direct repeats, one of which probably represents the *attB *site and partially overlaps with the anticodon and T loops of tRNA^Cys^.

### Genomic island PFGI-1

#### Location and integrase

Integrative conjugative elements (ICEs) are a rapidly growing class of strain-specific mosaic MGEs that can profoundly impact the adaptation and evolution of bacterial species [28]. ICEs vary in size from 10 to 500 kb, encode for mobility loci, and commonly exhibit anomalous G+C content and codon usage. Typical ICEs carry phage-like integrase genes that allow for site-specific integration, most often into tRNA genes, as well as plasmid-like replication and recombination functions and conjugative machinery that contributes to horizontal transfer. Finally, they often carry gene clusters encoding functions that are not essential for the host but that provide an advantage under particular environmental conditions. There is increasing evidence that ICEs derived from plasmids and encoding host-specific pathogenicity traits as well as traits essential for survival in natural habitats are widely distributed among members of the genus *Pseudomonas *[29-34].

**Figure 6:**
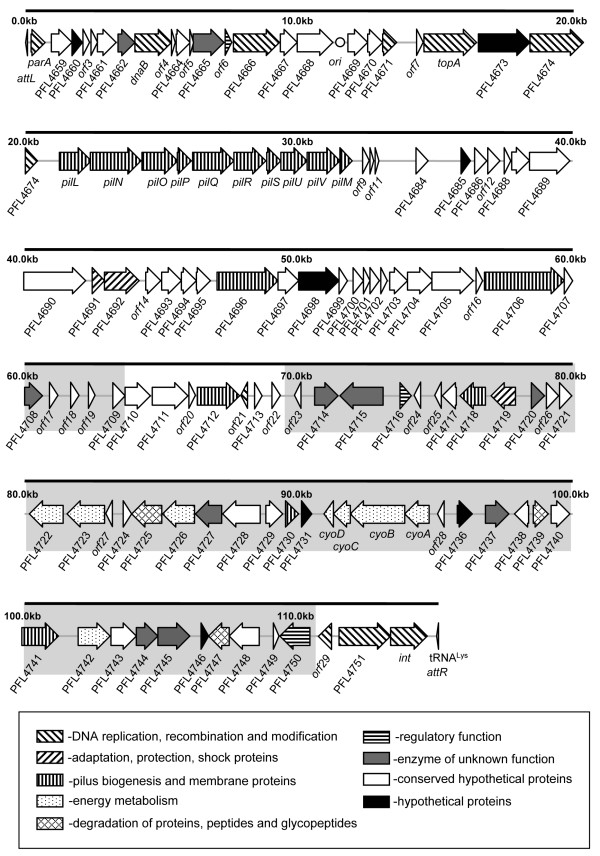
**Organization of genomic island PFGI-1**. Predicted open reading frames are shaded according to their category and their orientation is shown by arrows. DNA regions unique to *P. fluorescens *Pf-5 and not found in closely related GIs from other *Pseudomonas *spp. are indicated by grey shading.

**Figure 7:**
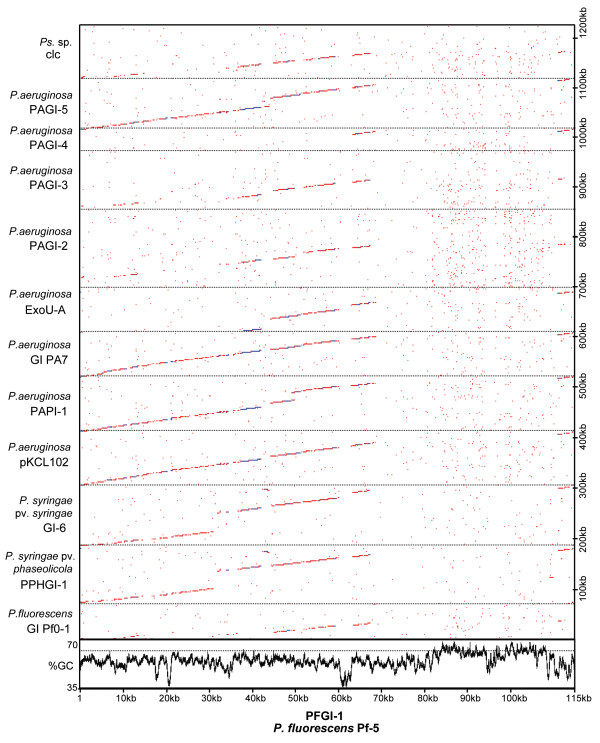
**Dot plot comparison of genomic island PFGI-1 with related genomic islands from other *Pseudomonas *spp**. Sequences of GI from *P. fluorescens *Pf0-1 [GenBank acc. CP000094; locus tags Pfl_O1_2993 through Pfl_O1_R50], PPHGI-1 from *P. syringae *pv. *phaseolicola *1302A [33], GI-6 from *P. syringae *pv. *syringae *B728a [36], pKCL102 from *P. aeruginosa *C [30], PAPI-1 from *P. aeruginosa *UCBPP-PA14 [32], GI from *P. aeruginosa *PA7 [GenBank acc. CP000744; locus tags PSPA7_4437 through PSPA7_4531], ExoU-A island from *P. aeruginosa *6077 [31], PAGI-2 and PAGI-4 from *P. aeruginosa *C [29], PAGI-3 from *P. aeruginosa *SGM17M [29], PAGI-5 from *P. aeruginosa *PSE9 [GenBank acc. EF611301], and *clc *element from *Pseudomonas *sp. B13 [34] were concatenated and aligned with PFGI-1 using a dot plot function from OMIGA 2.0 with sliding window of 45 and hash value of 6. Lower panel shows a 500-bp sliding window plot of G+C content for PFGI-1 with dotted line tracing the average G+C content (63%) of Pf-5 genome.

*P. fluorescens *Pf-5 harbors a 115-kb mobile genomic island 01, or PFGI-1 (Fig. 6, see Additional file 9), that resembles a large self-transmissible plasmid and exemplifies the first large plasmid-derived MGE found in *P. fluorescens*. Of 96 putative PFGI-1 coding sequences (CDSs), 50 were classified as hypothetical or conserved hypothetical genes, and 55 were unique to Pf-5 and absent from the genomes of strains SBW25 and Pf0-1 (Fig. 7). PFGI-1 is integrated into the tRNA^Lys ^gene (one of two genomic copies) situated next to PFL_4754, a CDS with similarity to *exsB*. Interestingly, this region has conserved synteny and probably represents an integration "hot spot" for CGIs in *Pseudomonas *spp., since putative integrase genes also are found adjacent to *exsB *in *P. aeruginosa *UCBPP-PA14 [35], *P. putida *KT2440 [25], *P. syringae *pv. *syringae *B728a [36] and *P. syringae *pv. *phaseolicola *1448A [37]. PFGI-1 spans 115,118 bp and is flanked by 49-bp direct repeats that include 45 bp of the 3' end of tRNA^Lys ^and represent a putative *attB *site. A recent survey of phage and tRNA integration sites by Williams [38] revealed that sublocation of *attB *within a tRNA gene correlates with subfamilies of tyrosine recombinases. According to this classification, the putative *attB *site of PFGI-1 falls into class IA since it encompasses both the T and anticodon loops of tRNA^Lys^. The integration of PFGI-1 probably is controlled by a phage-like tyrosine integrase encoded by PFL_4752 located 335 bp upstream from tRNA^Lys^.

#### Genes involved in plasmid replication, recombination, conjugative transfer, and possible origin of PFGI-1

Whether PFGI-1 exists in strain Pf-5 or in any other *Pseudomonas *host as an episome is not known. However, the first two-thirds of PFGI-1 contain putative plasmid replication, partitioning and conjugation genes that are readily aligned at the DNA level with those from plasmid pKLC102 of *P. aeruginosa *C [30]. The putative origin of replication, *oriV*, is situated immediately upstream of PFL_4669 and spans about 1,100 bp. Plasmid origins of replication often contain arrays of specific ~20 bp repeats, called iterons, that serve as binding sites for the cognate replication initiator Rep protein and are involved in replication and partitioning [39,40]. In addition to plasmid-specific iterons, some plasmid origins contain A+T-rich repeats where host replication initiation factors bind and open DNA, as well as repeats serving as binding sites for the host DnaA initiator protein. The putative *oriV *from PFGI-1 exhibits typical features of a plasmid replication origin. The first half is A+T-rich and has four conserved direct repeats of a perfect 23-bp palindrome (5'-CTGAGTTCGGAATCCGAACTCAGT-3'). The second half is represented by a G+C-rich stretch that overlaps with the region between PFL_4668 and PFL_4669 and contains four conserved 46-bp direct repeats, each of which includes an imperfect 21-bp inverted repeat (5'-AGTGTTGTGGGCCACACCACT-3'). The putative *oriV *is flanked by genes encoding proteins involved in plasmid replication, recombination and segregation, including putative homologues of DnaB and ParB (PFL_4663 and PFL_4666), a nucleoid associated protein (PFL_4665), topoisomerase TopA (PFL_4672), a putative helicase (PFL_4674), a single-strand binding protein (PFL_4671), and a partitioning protein, ParA (Fig. 6). PFGI-1 does not encode a Rep protein, and it is not clear whether it replicates by a theta-type or strand displacement mechanism, although the latter has been suggested for pKLC102 [30]. Like some conjugative plasmids, PFGI-1 carries homologues of the stress-inducible genes *umuC *(PFL_4692) and *umuD *(PFL_4691), which encode a putative lesion bypass DNA polymerase and a related accessory protein, respectively. Such genes may be involved in plasmid DNA repair and *umuDC*-mediated mutagenesis, which could allow plasmids to adapt more quickly to new bacterial hosts [41].

PFGI-1 also contains a cluster of 10 genes, *pilLNOPQRSTUVM *(PFL_4675 through PFL_4683) (Fig. 6), that spans over 10 kb and closely resembles part of the *pil *region from the self-transmissible *E. coli *plasmid R64 [42]. In *E. coli*, these genes are involved in production of thin flexible sex pili required for mating and transfer of R64 in liquid media. The similarity between the *pil *clusters of R64 and PFGI-1 suggests that the latter encode mating pili rather than type IV pili involved in bacterial twitching motility, adherence to host cells, biofilm formation and phage sensitivity [43]. *P. fluorescens *Pf-5 has the capacity to produce type IV pili, and the corresponding biosynthetic genes are located in at least three clusters found outside of PFGI-1. The PFGI-1 *pil *cluster contains genes for pilin protein PilS (PFL_4680), prepilin peptidase PilU (PFL_4681), outer membrane protein PilN (PFL_4676), nucleotide binding protein PilQ (PFL_4678), integral membrane protein PilR (PFL_4679), and pilus adhesin PilV (PFL_4682). Unlike R64, PFGI-1 does not include a shufflon region that determines recipient specificity in liquid matings via generation of different adhesin types [42,44]. Finally, PFGI-1 carries genes encoding proteins that may be involved in conjugal DNA transfer. PFL_4696 and PFL_4706 encode for TraG-like coupling proteins that may function as membrane-associated NTPases, which during conjugation would mediate transport of DNA covalently linked to a putative relaxase protein (the product of PFL_4751).

Recent studies have demonstrated that ICEs are a major component of a flexible gene pool of different lineages of Gram-negative Proteobacteria [45-47]. Metabolically versatile members of the Pseudomonadaceae are no exception, with ICEs having been identified among strains of *P. aeruginosa *[29-32], *P. syringae *[36,48], and *P. fluorescens *[49]. Comparison of PFGI-1 with islands from other *Pseudomonas *spp. reveals at least six highly conserved gene clusters (Fig. 7). These regions include: genes encoding partitioning (*parA *and *parB*) and single-strand binding (*ssb*) proteins, pilot proteins (*traG*), relaxase (PFL_4751), integrase (*int*), the putative origin of replication, and at least 26 conserved hypothetical ORFs. The *pil *operon is another syntenic cluster shared by PFGI-1 and the pathogenicity islands pKCL102 and PAPI-1, and PPHGI-1 of *P. aeruginosa *and and GI-6 of *P. syringae*. These findings further confirm the results of a recent study by Mohd-Zain et al. [47], who compared the evolutionary history of 33 core genes in 16 GIs from different β- and γ-Proteobacteria and found that despite their overall mosaic organization, many genomic islands including those from *Pseudomonas *spp. share syntenic core elements and evolutionary origin.

#### Putative phenotypic traits encoded by PFGI-1

As a rule, ICEs carry unique genes that reflect the lifestyles of their hosts. In *P. aeruginosa *and *P. syringae*, ICEs encode pathogenicity factors that allow these bacteria to successfully colonize a variety of hosts, as well as metabolic, regulatory, and transport genes that most probably enable them to thrive in diverse habitats [29,30,32,33,36,50]. An unusual self-transmissible ICE, the *clc *element from the soil bacterium *Pseudomonas *sp. B13, enables its host to metabolize chlorinated aromatic compounds [34,46,51].

In PFGI-1, a unique ~35 kb DNA segment that is absent from pKLC102 and other closely related ICEs (Figs. 6 and 7) encodes "cargo" genes that are not immediately related to integration, plasmid maintenance or conjugative transfer. Some of these genes are present in a single copy and do not have homologues elsewhere in the Pf-5 genome. About half of PFGI-1 "cargo" genes also are strain-specific and have no homologues in genome of *P. fluorescens *Pf0-1.

How could genes encoded by PFGI-1 contribute to the survival of *P. fluorescens *Pf-5 in the rhizosphere? Some of them might facilitate protection from environmental stresses. For example, nonheme catalases similar to the one encoded by PFL_4719 (Fig. 6) are bacterial antioxidant enzymes containing a dimanganese cluster that catalyzes the disproportionation of toxic hydrogen peroxide into water and oxygen [52]. PFGI-1 also carries a putative cardiolipin synthase gene (PFL_4745) and a cluster of four genes, *cyoABCD *(PFL_4732 through PFL_4735), that encode components of a cytochrome *o *ubiquinol oxidase complex. In *P. putida*, cardiolipin synthase was implicated in adaptation to membrane-disturbing conditions such as exposure to organic solvents [53], whereas the cytochrome *o *oxidase complex was shown to be highly expressed under low-nutrient conditions such as those found in the rhizosphere, and to play a crucial role in a proton-dependent efflux system involved in toluene tolerance [54,55]. Finally, PFGI-1 cargo genes with predicted regulatory functions include a GGDEF-motif protein (PFL_4715), a two-component response regulator with a CheY domain (PFL_4716) and a sensor histidine kinase (PFL_4750). Although the exact role of these genes remains unknown, the GGDEF domain proteins represent an emerging class of bacterial regulators involved in the synthesis of bis-(3'-5')-cyclic dimeric GMP, a global signaling messenger [56], and two-component signal-transduction systems are widely employed by Gram-negative bacteria to sense changes in the environment and appropriately modulate the expression of certain genes [57].

### Genomic island PFGI-2

Genomic island 02, or PFGI-2, spans 16.8 kb and has an average G+C content of 51.5%. It is flanked by imperfect 51-bp direct repeats, one of which partially overlaps with tRNA^Leu^(6) and probably represents the *attB *site (see Additional file 10). Although *P. fluorescens *Pf-5 does not have a type III protein secretion pathway, approximately half of PFGI-2 (i.e. an 8.1-kb DNA segment spanning genes PFL_4977 to PFL_4980) closely resembles a gene cluster found in the exchangeable effector locus (EEL) of a tripartite type III secretion pathogenicity island (T-PAI) from the plant pathogen *P. viridiflava *strain ME3.1b [58] (see Additional file 10). Even the presence of a putative phage integrase gene (PFL_4977) (see Additional files 5 and 10) and integration into tRNA^Leu ^immediately downstream of the *tgt *and *queA *genes is typical of T-PAI islands from *P. viridiflava *[58] and *P. syringae *[59]. In addition to T-PAI-like genes, PFGI-2 contains a putative phage-related MvaT-like (PFL_4981) transcriptional regulator, a superfamily II helicase (PFL_4979), a putative nucleoid-associated protein (PFL_4983), and a putative nuclease (PFL_4984). None of the aforementioned homologues of PFGI-2 genes in *P. viridiflava *have been characterized experimentally to date, making in difficult to deduce the function, if any, of this genome region. It also is possible that PFGI-2 is inactive and simply represents a T-PAI-like remnant anchored in the Pf-5 chromosome.

### Transposons of *P. fluorescens *Pf-5

Unlike the genomes of other *Pseudomonas *spp., that of *P. fluorescens *Pf-5 is devoid of IS elements and contains only one CDS (PFL_2698) that appears to encode a full-length transposase. Three other transposase-like CDSs (PFL_1553, PFL_3795, and PFL_2699) found in the Pf-5 genome contain frameshifts or encode truncated proteins. PFL_2698 and PFL_2699 encode IS66-like transposases and are found within a large cluster (PFL_2662 through PFL_2716) of conserved hypothetical genes. Corrupted transposases encoded by PFL_1553 and PFL_3795 belong to the IS5 family and are associated with gene clusters encoding a putative filamentous hemagglutinin and prophage 06, respectively.

## Conclusion

Recent analyses have revealed that most sequenced bacterial genomes contain prophages formed when temperate bacteriophages integrate into the host genome [60]. In addition to genes encoding phage-related functions, many prophages carry non-essential genes that can dramatically modify the phenotype of the host, allowing it to colonize or survive in new ecological niches [60,61]. Our knowledge of the ecological role of *Pseudomonas *prophages is limited, but data from other bacterial species suggest ways in which prophage regions could affect the survival of *P. fluorescens *Pf-5 in natural habitats.

Temperate bacteriophages similar to those encoded by prophages 03 and 06 are capable of development through both lysogenic and lytic pathways, and the presence of prophages can protect the host from superinfection by closely related bacteriophages [60]. On the other hand, the lytic pathway ultimately results in phage-induced host cell lysis, and it has been reported that the presence of virulent bacteriophages can adversely affect rhizosphere-inhabiting strains of *P. fluorescens *[62-64]. Similarly bacteriophage tail-like bacteriocins such as the one encoded by prophage 01 are capable of killing both closely and more distantly related strains of bacteria, presumably through destabilization of the cell membrane [65-69].

Temperate bacteriophages and bacteriophage-like elements also are an important part of the bacterial flexible gene pool and actively participate in horizontal gene transfer [60,70]. Among the putative lysogenic conversion genes in *P. fluorescens *Pf-5 are two copies of *llpA*, located adjacent to prophages 01 and 04. These genes encode low-molecular weight bacteriocins resembling plant mannose-binding lectins that kill sensitive strains of *Pseudomonas *spp. via a yet-unidentified mechanism [71]. The fact that both *llpA *copies reside near prophage repressor genes, as well as the involvement of a *recA*-dependent SOS response in LlpA production by a different strain of *Pseudomonas *[72], suggests that the association of *llpA *genes with prophages is not accidental and that the prophages may be involved in the regulation of bacteriocin production in *P. fluorescens *Pf-5.

The analysis of MGEs revealed at least 66 CDSs not present in the original Pf-5 genome annotation (data are summarized in supplemental Tables). The bulk of these newly predicted CDSs fall in the category of conserved hypothetical genes of bacterial or phage origin. Predicted products of the remaining novel CDSs exhibit similarity to proteins of diverse enzymatic, regulatory, and structural functions and include a phage integrase, an ATP-dependent DNA ligase, an endonuclease, plasmid partitioning and stabilization proteins, a NADH-dependent FMN reductase, an acytransferase, a PrtN-like transcriptional regulator, a Com-like regulatory protein, a P-pilus assembly and an integral membrane protein.

Taken together, the analyses of six prophage regions and two GIs in the Pf-5 genome indicate that these structures have evolved via exchange of genetic material with other *Pseudomonas *spp. and extensive recombination. Transposition is unlikely to have played a major role in this evolution, as the genome of Pf-5 is nearly devoid of transposons and IS elements that are common in certain other *Pseudomonas *genomes. Most of the 260 kb comprising MGEs in the Pf-5 genome is similar to corresponding regions in the genomes of other *Pseudomonas *spp. Nevertheless, the MGEs also include regions unique to the Pf-5 genome that could contribute to the bacterium's fitness in the soil or rhizosphere.

## Methods

### Strains and plasmids

Wild type variants of *P. fluorescens *Pf-5 [5], *P. fluorescens *SBW25 [73], and *P. fluorescens *Q8r1-96 [74] were used in the study. *Pseudomonas *strains were grown at 28°C in King's B medium [75], while *E. coli *strains were grown in LB [76] or 2xYT [76] at 25°C or 37°C. When appropriate, antibiotic supplements were used at the following concentrations: tetracycline, 12.5 μg/ml; chloramphenicol, 35 μg/ml; and ampicillin, 100 μg/ml.

### DNA manipulations and sequence analyses

Plasmid DNA isolation, restriction enzyme digestion, agarose gel electrophoresis, ligation, and transformation were carried out using standard protocols [76]. All primers were developed with Oligo 6.65 Software (Molecular Biology Insights, West Cascade, Colo.), and routine PCR amplifications were performed with *Taq *DNA polymerase (Promega, Madison, Wisc.) according to the manufacturer's recommendations.

Sequencing of prophage 01 from *P. fluorescens *Q8r1-96 was carried out essentially as described by Mavrodi et al. [77]. Briefly, the Q8r1-96 gene library was screened by PCR with oligonucleotide primers col1 (5' GCT GCT GGG CAA TGG TAA CAC 3') and col2 (5' CTG CCG ACT GCT CAC CTA TC 3') and a positive cosmid clone was shotgun sequenced by using the EZ::TN™ <Kan-2> transposition system (Epicentre Technologies, Madison, Wisc.). DNA sequencing was carried out by using an ABI PRISM BigDye Terminator Cycle Sequencing Ready Reaction Kit (Applied Biosystems, Foster City, Calif.), and sequence data were compiled and analyzed with Vector NTI 9.1.0 (Invitrogen Corp., Carlsbad, Calif.) and OMIGA 2.0 (Accelrys, San Diego, Calif.) software packages. Database searches for similar protein sequences were performed using the NCBI's BLAST network service, and searches against PROSITE, Profile, HAMAP, and Pfam collections of protein motifs and domains were carried out by using the MyHits Internet engine [78]. Signal peptides were predicted with SignalP v. 3.0. [79]. The nucleotide sequence of prophage 01 from *P. fluorescens *Q8r1-96 has been deposited in GenBank under accession number EU982300.

### DNA hybridization

The 3.12-kb prophage 01 probe was amplified by PCR from *P. fluorescens *Q8r1-96 genomic DNA with the oligonucleotide primers orf11-1 (5' CAT TCG TGT GCC GCT GTT CTA 3') and orf14-2 (5' TGA CCA GGC GAA CAG CGT CTG 3'). The 1.79-kb *P. fluorescens *SBW25-specific prophage 01 probe was amplified from genomic DNA of SBW25 with oligonucleotides SBW3 (5' GAA CTC ACC AGC GTC CTT AAC 3') and SBW4 (5' GGG CAG CTC CTT GGT GAA GTA 3'). Amplification was carried out with Expand Long DNA polymerase (Roche Applied Science, Indianapolis, IN) according to manufacturer's recommendations. The cycling program included a 2-min initial denaturation at 94°C followed by 25 cycles of 94°C for 10 sec, 60°C for 30 sec, and 68°C for 3 min. Amplified DNA was gel-purified with a QIAEX II gel extraction kit (Qiagen, Santa Clarita, Calif.) and labeled with the Biotin High Prime System (Roche Applied Science). Genomic DNA was purified using a cetyltrimethylammonium bromide miniprep protocol [76], digested with *Eco*RI and *Pst*I, separated by electrophoresis in a 0.8% agarose gel, and transferred onto a BrightStar-Plus nylon membrane (Ambion, Inc., Austin, TX) in 0.4 M NaOH. The membranes were pre-hybridized and hybridized at 58°C in a solution containing 5 × SSC [80], 4 × Denhardt's solution [80], 0.1% SDS, and 300 μg per ml of denatured salmon sperm DNA (Sigma). After hybridization, the membranes were washed twice in 2 × SSC, 0.1% SDS at room temperature, twice in 0.2 × SSC, 0.1% SDS at room temperature, and once in 0.2 × SSC, 0.1% SDS at 60°C.

### Lytic assays

Full-length *hol *genes from strains Pf-5 and Q8r1-96 were amplified by using KOD Hot Start DNA polymerase (Novagen, Inc.) and oligonucleotide primer pairs holupPf5 (5' AGG GAC CTC TAG AAA CAT CGT TA 3') – holowPf5 (5' TTT TGG ATC CGG TGA GTC AAG GCT G 3') and hol-xba (5' GAC CAG TCT AGA CAT GCT CAT CA 3') – hol-low (5' TTT TGG ATC CGC GGT ATC GCT T 3'), respectively. Full-length *lys *genes from Pf-5 and Q8r1-96 were amplified by using primer sets lysupPf5 (5' CGC CAT TCT AGA TTA CTG AAC AA 3') – lyslowPf5 (5' TTT TGG ATC CGC AGG ACC TTC AGA C 3') and lysQ8-up (5' CGG ACA TCT AGA ATC ATG CAC TTG 3') – tail13 (5' GCC GCT TGG GTG ATT TGA TT 3'), respectively. The cycling program included a 2-min initial denaturation at 94°C followed by 35 cycles of 94°C for 15 sec, 59°C for 30 sec, and 68°C for 1 min, and a final extension at 68°C for 3 min. PCR products were gel-purified and cloned into the *Sma*I site of the plasmid vector pCR-Blunt (Invitrogen) under the control of the T7 promoter. The resultant plasmids were single-pass sequenced to confirm the integrity of cloned genes and electroporated into *E. coli *Rosetta/pLysS (Novagen) with a Gene Pulser II system (Bio-Rad Laboratories, Hercules, Calif.). Plasmid-bearing *E. coli *clones were selected overnight on LB agar supplemented with ampicillin and chloramphenicol, and suspended in 2xYT broth supplemented with antibiotics to give an OD_600 _of 0.1. After incubation with shaking for one hour at room temperature, gene expression was induced in the broth cultures with 3 mM IPTG. The induced cultures were incubated with shaking for another 5 hours and the cell density was monitored by measuring OD_600 _every 30 min. To disrupt cell membranes in endolysin-expressing cultures, a drop of chloroform was added after four hours of induction. Two independent repetitions were performed with each strain.

## Authors' contributions

DVM was responsible for conception of the study, experimental design, data collection, and analysis. LST, ITP and JEL participated in data analysis and preparation of the manuscript.

## Supplementary Material

Additional file 1 **Sequence analysis of prophage 01 of *P. fluorescens *Pf-5.** Table containing annotation of mobile genetic element prophage 01 in the genome of *Pseudomonas fluorescens *Pf-5. The following information is provided for each open reading frame: locus tag number, gene name, genome coordinates, length and molecular weight of encoded protein, sequence of putative ribosome binding site, description of the closest GenBank match plus blast E-value, list of functional domains and predicted function. Click here for file

Additional file 2 **Sequence analysis of prophage 01 of *P. fluorescens *Q8r1-96.** Table containing annotation of mobile genetic element prophage 01 in the genome of *Pseudomonas fluorescens *Q8r1-96. The following information is provided for each open reading frame: locus tag number, gene name, genome coordinates, length and molecular weight of encoded protein, sequence of putative ribosome binding site, description of the closest GenBank match plus blast E-value, list of functional domains and predicted function. Click here for file

Additional file 3 **Sequence analysis of prophage 03 of *P. fluorescens *Pf-5.** Table containing annotation of mobile genetic element prophage 03 in the genome of *Pseudomonas fluorescens *Pf-5. The following information is provided for each open reading frame: locus tag number, gene name, genome coordinates, length and molecular weight of encoded protein, sequence of putative ribosome binding site, description of the closest GenBank match plus blast E-value, list of functional domains and predicted function. Click here for file

Additional file 4 **Sequence analysis of prophage 06 of *P. fluorescens *Pf-5.** Table containing annotation of mobile genetic element prophage 06 in the genome of *Pseudomonas fluorescens *Pf-5. The following information is provided for each open reading frame: locus tag number, gene name, genome coordinates, length and molecular weight of encoded protein, sequence of putative ribosome binding site, description of the closest GenBank match plus blast E-value, list of functional domains and predicted function. Click here for file

Additional file 5 **Sequence analysis of putative integrase genes from *P. fluorescens *Pf-5.** Table containing annotation of putative integrase genes present in the genome of *Pseudomonas fluorescens *Pf-5. The following information is provided for each open reading frame: locus tag number, gene name, genome coordinates, length and molecular weight of encoded protein, sequence of putative ribosome binding site, description of the closest GenBank match plus blast E-value, list of functional domains and predicted function. Click here for file

Additional file 6 **Sequence analysis of prophage 02 of *P. fluorescens *Pf-5.** Table containing annotation of mobile genetic element prophage 02 in the genome of *Pseudomonas fluorescens *Pf-5. The following information is provided for each open reading frame: locus tag number, gene name, genome coordinates, length and molecular weight of encoded protein, sequence of putative ribosome binding site, description of the closest GenBank match plus blast E-value, list of functional domains and predicted function. Click here for file

Additional file 7 **Sequence analysis of prophage 04 of *P. fluorescens *Pf-5.** Table containing annotation of mobile genetic element prophage 04 in the genome of *Pseudomonas fluorescens *Pf-5. The following information is provided for each open reading frame: locus tag number, gene name, genome coordinates, length and molecular weight of encoded protein, sequence of putative ribosome binding site, description of the closest GenBank match plus blast E-value, list of functional domains and predicted function. Click here for file

Additional file 8 **Sequence analysis of prophage 05 of *P. fluorescens *Pf-5.** Table containing annotation of mobile genetic element prophage 05 in the genome of *Pseudomonas fluorescens *Pf-5. The following information is provided for each open reading frame: locus tag number, gene name, genome coordinates, length and molecular weight of encoded protein, sequence of putative ribosome binding site, description of the closest GenBank match plus blast E-value, list of functional domains and predicted function. Click here for file

Additional file 9 **Sequence analysis of island 01 of *P. fluorescens *Pf-5.** Table containing annotation of mobile genetic element island 01 in the genome of *Pseudomonas fluorescens *Pf-5. The following information is provided for each open reading frame: locus tag number, gene name, genome coordinates, length and molecular weight of encoded protein, sequence of putative ribosome binding site, description of the closest GenBank match plus blast E-value, list of functional domains and predicted function. Click here for file

Additional file 10 **Sequence analysis of island 02 of *P. fluorescens *Pf-5.** Table containing annotation of mobile genetic element island 02 in the genome of *Pseudomonas fluorescens *Pf-5. The following information is provided for each open reading frame: locus tag number, gene name, genome coordinates, length and molecular weight of encoded protein, sequence of putative ribosome binding site, description of the closest GenBank match plus blast E-value, list of functional domains and predicted function. Click here for file
