# Supplementary material for: Mobile genetic elements in the genome of the beneficial rhizobacterium Pseudomonas fluorescens Pf-5
Source: BMC Microbiol. 2009 Jan 13;9:8. doi: 10.1186/1471-2180-9-8 (PMC2647930; doi:10.1186/1471-2180-9-8)
Supplement: Additional file 1 — Sequence analysis of prophage 01 of P. fluorescens Pf-5. Table containing annotation of mobile genetic element prophage 01 in the genome of Pseudomonas fluorescens Pf-5. The following information is provided for each open reading frame: locus tag number, gene name, genome coordinates, length and molecular weight of encoded protein, sequence of putative ribosome binding site, description of the closest GenBank match plus blast E-value, list of functional domains and predicted function. [file 1471-2180-9-8-S1.pdf]

Additional file 1 – Sequence analysis of prophage 01 of *P. fluorescens* Pf-5 genome

CDS	Gene name	Position	Protein length (residues)	Protein MW (kDa)	Putative ribosome binding site	Closest protein of phage or plasmid origin (accession no.), blastp E-value[a]	Overall closest protein (accession no.), blastp E-value[a]	Predicted function (functional domains)
PFL_1210	*prtR*	1386136..1386882	266	30.036	GTGTT	Pyocin repressor PrtR from *Pseudomonas aeruginosa* (Q06553), 5e-35	Hypothetical protein Pfl01_1135 from *Pseudomonas fluorescens* Pf0-1 (ABA72878); 2e-129	Prophage repressor, Cro/CI family; HTH domain (COGs 1396, 2932, 4800)
PFL_1211	*hol*	1387400..1387750	116	12.379	GGAG	R2/F2 pyocin-specific holin from *Pseudomonas aeruginosa* (BAA83151), 2e-17	Hypothetical protein Pfl01_1137 from *Pseudomonas fluorescens* Pf0-1 (ABA72880);7e-50	Holin
PFL_1212	PFL_1212	1387843..1388166	107	11.664	GCAGG	PRF23 from R2/F2 pyocin region from *Pseudomonas aeruginosa* (BAA83167), 8e-18	Putative protein D (AAO56868), *P. syringae pv. tomato* DC3000, 1e-25	Tail protein D
PFL_1213	PFL_1213	1388216..1389352	378	40.966	AGGAG	None detected	Hypothetical protein YvbX from *Bacillus subtilis* (NP_391282), 2e-40	Putative peptidoglycan hydrolase (COGs 3325, 3858)
PFL_1214	PFL_1214	1389510..1390091	193	20.897	AGGAG	Hypothetical protein Sb11 from *Salmonella typhimurium* phage ST64B (NP_700384), 1e-19	Hypothetical protein Psyr4420 from *P. syringae pv. tomato* DC3000 (ZP_00128092), 9e-47	Hypothetical phage protein
PFL_1215	PFL_1215	1390088..1390270	60	6.345	GGAGA	Hypothetical protein Orf10 from *Shigella flexneri* phage V (AAL89419), 1e-06	Hypothetical protein Pfl01_1153 from *Pseudomonas fluorescens* Pf0-1 (ABA72896);2e-17	Hypothetical phage protein
PFL_1216	PFL_1216	1390270..1391766	498	52.810	AGGAG	Tail sheath protein Sb13 from *Salmonella typhimurium* phage ST64B	Hypothetical protein Pfl01_1154 from *Pseudomonas*	Tail sheath protein (COG4386)

						(NP_700386), 1e-131	*fluorescens* Pf0-1 (ABA72897);0.0	
PFL_1217	PFL_1217	1391833..1392180	115	12.360	GGAG	Tail tube protein Sb14 from *Salmonella typhimurium* phage ST64B (NP_700387), 3e-16	Hypothetical protein Pfl01_1155 from *Pseudomonas fluorescens* Pf0-1 (ABA72898);1e-54	Tail tube protein
PFL_1218	PFL_1218	1392177..1392473	98	10.538	GAAGG	Hypothetical protein Orf13 from *Shigella flexneri* phage V (NP_599045), 3e-11	Hypothetical protein Pfl01_1156 from *Pseudomonas fluorescens* Pf0-1 (ABA72899);1e-45	Hypothetical protein
PFL_1219	PFL_1219	1392604..1394202	532	55.965	AGGTG	Tail protein Orf14 from *Shigella flexneri* phage V (NP_599046), 3e-16	Hypothetical protein Pfl01_1157 from *Pseudomonas fluorescens* Pf0-1 (ABA72900);0.0	Tail tape measure protein (COG5412)
PFL_1220	PFL_1220	1394192..1395436	414	45.777	GGAGG	Tail/DNA circulation protein Orf15 from *Shigella flexneri* phage V (NP_599047), 5e-36	Hypothetical protein Pfl01_1158 from *Pseudomonas fluorescens* Pf0-1 (ABA72901);1e-157	Tail/DNA circulation protein (COG4228)
PFL_1221	PFL_1221	1395440..1396480	346	37.632	GGAG	Tail protein Orf15 from *Shigella flexneri* phage V (NP_599048), 2e-55	Hypothetical protein Pfl01_1159 from *Pseudomonas fluorescens* Pf0-1 (ABA72902);8e-146	Tail protein P (COG4379)
PFL_1222	PFL_1222	1396590..1397096	168	17.601	AAGGA	Tail protein Orf17 from *Shigella flexneri* phage V (NP_599049), 1e-18	Hypothetical protein Pfl01_1160 from *Pseudomonas fluorescens* Pf0-1 (ABA72903);1e-76	Baseplate assembly protein (COG4384)
PFL_1223	PFL_1223	1397099..1397497	132	15.415	GGAGG	Tail protein Orf18 from *Shigella flexneri* phage V (NP_599050), 9e-15	Hypothetical protein Pfl01_1161 from *Pseudomonas fluorescens* Pf0-1 (ABA72904);8e-49	Tail protein (COG4381)

PFL_1224	PFL_1224	1397487..1398527	346	36.968	AGGTG	Tail protein Orf19 from *Shigella flexneri* phage V (NP_599051), 7e-58	Hypothetical protein Pfl01_1162 from *Pseudomonas fluorescens* Pf0-1 (ABA72905);4e-152	Tail protein (COG3299)
PFL_1225	PFL_1225	1398515..1399114	199	22.390	GGAGG	Tail protein Orf20 from *Shigella flexneri* phage V (NP_599052), 4e-08	Hypothetical protein Pfl01_1163 from *Pseudomonas fluorescens* Pf0-1 (ABA72906);7e-87	Tail protein (COG3778)
PFL_1226	PFL_1226	1399130..1400239	369	38.720	GAGG	Side tail fibre protein StfE from *E.coli* lambdoid prophage e14 (P33227), 6e-16	Hypothetical protein PSEEN4159 from *Pseudomonas entomophila* L48 (YP_609632);1e-57	Tail fibre protein (COG5301)
1[b]	*orf1*	1400236..1400613	125	14.609	GGAG	Hypothetical protein Orf21 from *Pseudomonas aeruginosa* phage φCTX (BAA36248), 4e-11	Hypothetical protein Pfl01_1169 from *Pseudomonas fluorescens* Pf0-1 (ABA72912);7e-87	Hypothetical phage protein
PFL_1227	*lys*	1400664..1401227	187	20.881	AAACA	R2/F2 pyocin-specific lysozyme from *Pseudomonas aeruginosa* (BAA83168), 2e-49	Hypothetical protein Pfl01_1172 from *Pseudomonas fluorescens* Pf0-1 (ABA72915);1e-89	Endolysin (COG3179)
PFL_1228	PFL_1228	1401335..1401850	171	18.042	AGGAG	Hypothetical protein RPRSA1_gp15 from *Ralstonia* phage phiRSA1 (YP_001165264), 6e-21	Conserved hypothetical protein Bcenmc03_0198 from *Burkholderia cenocepacia* MC0-3 (ACA89378);1e-28	Conserved hypothetical phage protein
2[b]	*orf2*	1401912..1402109	65	7.246	GGATA	Translational regulator Com from *Pseudomonas* phage B3 (AAQ13965), 2e-08	Conserved hypothetical protein PA14_14540 from *Pseudomonas aeruginosa* UCBPP-PA14 (ABJ13088), 2e-16	Com-like phage protein (COG4416)
PFL_1229	*llpA1*	1402010..1402960	280	31.032	AGGTA	None detected	Bacteriocin putidacin L1	Lectin-like

(AAM95702), *Pseudomonas* sp. BW11M1, 9e-61	bacteriocin putidacin

[a] Only expectation values of $1e^{-05}$ and below were considered as significant matches during BLAST database searches; [b] open reading frames not present in the original genome annotation.
