# Supplementary material for: Mobile genetic elements in the genome of the beneficial rhizobacterium Pseudomonas fluorescens Pf-5
Source: BMC Microbiol. 2009 Jan 13;9:8. doi: 10.1186/1471-2180-9-8 (PMC2647930; doi:10.1186/1471-2180-9-8)
Supplement: Additional file 2 — Sequence analysis of prophage 01 of P. fluorescens Q8r1-96. Table containing annotation of mobile genetic element prophage 01 in the genome of Pseudomonas fluorescens Q8r1-96. The following information is provided for each open reading frame: locus tag number, gene name, genome coordinates, length and molecular weight of encoded protein, sequence of putative ribosome binding site, description of the closest GenBank match plus blast E-value, list of functional domains and predicted function. [file 1471-2180-9-8-S2.pdf]

Additional file 2 – Sequence analysis of prophage 01 of *P. fluorescens* Q8r1-96

CDS	Gene name	Position	Protein length (residues)	Protein MW (kDa)	Putative ribosome binding site	Closest protein of phage or pyocin origin (accession no.), blastp E-value[a]	Overall closest protein (accession no.), blastp E-value[a]	Predicted function (functional domains)
1	*orf1*	52..405 (-)	117	12.794	AAGGA	Hypothetical protein from *Pseudomonas* sp. MIS38 (BAF40425); 7e-22	Hypothetical protein from Pfl01_1963 from *P. fluorescens* Pf0-1 (ABA73706); 2e-20	Conserved hypothetical protein
2	*prtR*	906..1709	267	30.178	CATTG	Negative regulator PrtR of pyocin genes from *P. aeruginosa* PAO1 (BAA02198); 5e-41	Possible phage repressor of Cro/CI family from *P. fluorescens* Pf0-1 (ABA72878); 1e-136	Prophage repressor, Cro/CI family; HTH domain (COGs 2932, 4800, 4800)
3	*orf3*	2025..2333	102	11.342	GAGG	Com translational regulator from *Pseudomonas* phage B3 (AAQ13965); 2e-06	Hypothetical protein Psyr_4601 from *P. syringae pv. syringae* B728a, , (AAY39631); 1e-12	Mu-like prophage translational regulator Com (COG4416)
4	*orf4*	2354..2998	214	22.795	GAGG	None detected	Hypothetical protein Ava_C0051 from *Anabaena variabilis* ATCC29413 (ABA25140); 2e-14	Conserved hypothetical protein
5	*hol*	3046..3396	116	12.447	GGAAG	Holin from pyocin region from *P. aeruginosa* PAO1 (BAA83136); 9e-27	Putative holin PFL_1211 (AAY90498) from *P. fluorescens* Pf-5; 1e-51	Holin
6	*cma*	3621..4436	271	29.433	AGGAA	None detected	Hypothetical protein EXA13 from *P. aeruginosa* 6077 (ABD94622); 6e-24	Putative bacteriocin with similarity to colicin M
7	*orf7*	4553..4990	145	15.572	AGGA	None detected	None detected	Hypothetical protein
8	*orf8*	5859..6056	65	7.313	GAGG	None detected	None detected	Hypothetical

								protein
9	*orf9*	6285..6869	194	21.236	GGAG	Hypothetical protein Sb11 from *Salmonella typhimurium* phage ST64B (AAL25890), 4e-21	Hypothetical protein Pfl01_1152 from *P. fluorescens* Pf0-1 (ABA72895); 7e-83	Hypothetical bacteriophage protein
10	*orf10*	6866..7048	60	6.488	GGAG	Hypothetical protein Orf10 from *Shigella flexneri* phage V (AAL89419), 1e-05	Hypothetical protein PFL_1215 (AAY90502) from *P. fluorescens* Pf-5; 2e-21	Hypothetical bacteriophage protein
11	*orf11*	7048..8544	498	52.980	GGAG	Tail sheath protein Sb13 from *Salmonella typhimuriu*m phage ST64B (AAL25892), 1e-151	Mu-like prophage tail sheath protein gpL (Pfl01_1154) from *P. fluorescens* Pf0-1 (ABA72897); 0.0	Tail sheath protein (COG4386)
12	*orf12*	8611..8958	115	12.411	GAAGG	Tail tube protein gpM Sb14 from *Salmonella typhimurium* phage ST64B (AAL25893), 2e-16	Hypothetical protein Pfl01_1155 from *P. fluorescens* Pf0-1 (ABA72898); 1e-57	Tail tube protein gpM
13	*orf13*	8955..9251	98	10.474	AAGGGGA	Hypothetical protein Orf13 from *Shigella flexneri* phage V (AAL89422), 2e-11	Hypothetical protein Pfl01_1157 from *P. fluorescens* Pf0-1 (ABA72900); 1e-44	Small phage tail protein E
14	*orf14*	9382..10956	524	54.549	GGTG	Tail protein Orf14 from *Shigella flexneri* phage V (AAL89423), 2e-16	Hypothetical protein Pfl01_1157 from *P. fluorescens* Pf0-1 (ABA72900); 2e-145	Phage tail tape measure protein (COG5412)
15	*orf15*	10943..12181	412	45.153	GGAGG	Tail DNA circulation protein Orf15 from *Shigella flexneri* phage V (AAL89424), 5e-37	Mu-like prophage DNA circulation protein PFL_1220 from *P. fluorescens* Pf-5 (AAY90507); 2e-180	Phage DNA circulation protein (COG4228)
16	*orf16*	12185..13225	346	37.735	GGAG	Tail protein P Orf16 from *Shigella flexneri* phage V (AAL89425), 6e-57	Mu-like prophage tail protein gpP (Pfl01_1159) from *P. fluorescens* Pf0-1 (ABA72902); 6e-163	Phage tail protein gpP (COG4379)
17	*orf17*	13490..13999	169	17.780	AAGGA	Putative baseplate assembly	Mu-like prophage	Baseplate

						protein Gp45 from *E. coli* phage P27 (CAC83568), 2e-20	protein gp45 (Pfl01_1160) from *P. fluorescens* Pf0-1 (ABA72903); 1e-82	assembly protein Gp45 (COG4384)
18	*orf18*	13999..14397	132	15.308	GGAGG	Phage protein gp46 from *Shigella flexneri* bacteriophage V (AAL89427), 9e-20	Mu-like prophage protein gp46 (Pfl01_1161) from *P. fluorescens* Pf0-1 (ABA72904); 1e-57	Phage protein gp46 (pfam07409)
19	*orf19*	14387..15427	346	36.701	GGTG	Tail protein gp47 from *Shigella flexneri* phage V (AAL89428), 2e-57	Phage FluMu protein gp47 (PFL_1224) from *P. fluorescens* Pf-5 (AAY90511); 3e-165	Tail protein gp47 (COG3299)
20	*orf20*	15415..16014	199	22.181	GGAGG	Protein of unknown function Sb23 from *Salmonella typhimurium* phage ST64B (AAL25901), 1e-08	Putative phage tail protein Pfl01_1163 from *P. fluorescens* Pf0-1 (ABA72906); 6e-96	Hypothetical bacteriophage protein (COG3778)
21	*orf21*	16026..17180	384	40.305	GGAG	None detected	Hypothetical protein Pfl01_1164 from *P. fluorescens* Pf0-1 (ABA72907); 1e-155	Putative tail fiber protein
22	*orf22*	17177..17686	169	18.503	GGAG	Tail fiber assembly protein gp25 from *Ralstonia* phage phiRSA1 (BAF52402), 1e-21	Putative tail fiber assembly protein Pfl01_1165 from *P. fluorescens* Pf0-1 (ABA72908); 4e-46	Putative tail fiber protein
23	*orf23*	17714..18808	364	38.787	GAGAGA	Protein gp32 from *Burkholderia* phage phiE202 (AB060734), 1e-13	Hypothetical protein Pfl01_1166 from *P. fluorescens* Pf0-1 (ABA72909); 2e-157	Phage tail collar protein
24	*orf24*	18816..19376	186	20.974	GAGG	Putative tail fiber assembly protein from *E. coli* O157:H7 prophage CP-933X (AAG56009), 5e-10	Hypothetical protein Pfl01_1167 from *P. fluorescens* Pf0-1 (ABA72910); 5e-69	Conserved hypothetical protein
25	*orf25*	19539..20399	286	29.675	GCAGG	Putative tail fiber protein of *Burkholderia* phage Bups phi1 (ABY40547), 1e-07	Putative tail fiber assembly protein Pfl01_1168 from *P.*	Putative tail fiber assembly protein

							fluorescens Pf0-1 (ABA72911); 7e-41	
26	*orf26*	20413..20844	143	16.383	GGAGG	Hypothetical p23 protein of *Pseudomonas* phage phiCTX (BAA36258), 2e-21	Hypothetical protein Pfl01_1169 from *P. fluorescens* Pf0-1 (ABA72912); 1e-21	Conserved hypothetical phage protein
27	*lys*	20869..21432	187	20.950	GATG	Lytic enzyme (BAA83168) from pyocin region of *P. aeruginosa* PAO1, 9e-36	Putative lytic enzyme Pfl01_1171from *P. fluorescens* Pf0-1 (ABA72915); 7e-85	Endolysin (COG3179)
28	*orf28*	21414..21950	178	19.308	GGTG	None detected	Putative phage protein PFL_3742 from *P. fluorescens* Pf-5 (AAY93007); 2e-52	Conserved hypothetical protein
cinA	*cinA*	22022..22522	166	17.570	GGAG	None detected	CinA-like protein Pfl01_1174 from *P. fluorescens* Pf0-1 (ABA72917); 7e-77	Competence damage-inducible protein A (COG1546)
recA	*recA*	22606..22689	na	na	GAGGA	None detected	Recombinase A protein from *P. fluorescens* Pf0-1 (ABA72918); 3e-18	Recombinase A

[a] Only expectation values of $1e^{-05}$ and below were considered as significant matches during BLAST database searches.
