# Supplementary material for: Mobile genetic elements in the genome of the beneficial rhizobacterium Pseudomonas fluorescens Pf-5
Source: BMC Microbiol. 2009 Jan 13;9:8. doi: 10.1186/1471-2180-9-8 (PMC2647930; doi:10.1186/1471-2180-9-8)
Supplement: Additional file 3 — Sequence analysis of prophage 03 of P. fluorescens Pf-5. Table containing annotation of mobile genetic element prophage 03 in the genome of Pseudomonas fluorescens Pf-5. The following information is provided for each open reading frame: locus tag number, gene name, genome coordinates, length and molecular weight of encoded protein, sequence of putative ribosome binding site, description of the closest GenBank match plus blast E-value, list of functional domains and predicted function. [file 1471-2180-9-8-S3.pdf]

Additional file 3 – Sequence analysis of prophage 03 of *P. fluorescens* Pf-5

CDS	Gene name	Position	Protein length (residues)	Protein mol weight (kDa)	Putative ribosome binding site	Closest protein of phage or plasmid origin (accession no.), blastp E-value [a]	Other similar proteins (accession no.), blastP E-value [a]	Predicted function (functional domains)
PFL_1976	*int2*	2207060..2208202	380	43.927	GTAG	Shufflon-specific recombinase from plasmid ColIb-P9 (AAZ05392), 4e-21	Phage integrase family protein PputGB1_1711 from *Pseudomonas putida* GB-1 (YP_001667950), 0.0	Putative phage integrase
1[b]	*orf1*	2208171..2208452 (-)	93	10.325	GGAG	Transcriptional regulator PrtN from *Pseudomonas aeruginosa* pyocin (Q06552); $5e^{-05}$	Conserved hypothetical protein PputGB1_1711 from *Pseudomonas putida* GB-1 (ABY97615), 3e-36	Putative phage-related positive regulator
PFL_1977	PFL_1977	2208449..2208922 (-)	157	16.913	GGAG	None detected	None detected	Hypothetical protein
2[b]	*orf2*	2209446..2211179 (-)	176	19.300	AGGAG	Hypothetical protein gp37 from *Burkholderia phage* BcepC6B (AAT38396); 1e-15	Hypothetical protein PA2G_00782 from *Pseudomonas aeruginosa* 2192 (EAZ57582); $2e^{-13}$	Conserved hypothetical phage protein
PFL_1978	PFL_1978	2209446..2211179 (-)	577	63.298	GGAG	Hypothetical protein gp41 from *Pseudomonas aeruginosa* phage CTX (NP_490641); 0.0	Hypothetical protein Orf27 from *Pseudomonas putida* plasmid pWWO (NP_542819)*;* 0.0	Conserved hypothetical protein (COG0270)
PFL_1979	PFL_1979	2211169..2211507 (-)	112	12.047	AGGAG	None detected	Hypothertical protein PP3030 from *Pseudomonas putida* KT2440 (NP_745174); $4e^{-10}$	Conserved hypothetical protein
PFL_1980	PFL_1980	2211507..2211728 (-)	73	8.368	AGGAG	None detected	None detected	Hypothetical protein

PFL_1981	PFL_1981	2211725..2212144 (-)	139	15.210	GGAG	None detected	Hypothetical protein PSPTO3426 from *Pseudomonas syringae* pv. *tomato* DC3000 (NP_793209), 6e^{-24}	Conserved hypothetical protein
PFL_1982	*prtN*	2212189..2212515 (-)	108	12.260	GCAAG	Transcriptional regulator PrtN from *Pseudomonas aeruginosa* pyocin (Q06552); 5e^{-05}	Putative transcriptional regulator PSPTO3425 from *Pseudomonas syringae* pv. *tomato* DC3000 (NP_793208), 3e^{-40}	Putative phage-related positive regulator
PFL_1983	PFL_1983	2212674..2212958 (-)	94	10.986	GGAG	None detected	Putative DNA-binding Roi-like protein PSPTO3423 from *Pseudomonas syringae* pv. *tomato* DC3000 (NP_793206); 1e^{-33}	Conserved hypothetical phage protein
PFL_1984	PFL_1984	2212968..2213552 (-)	194	21.429	GGAG	None detected	Hypothetical protein PSPTO3422 from *Pseudomonas syringae* pv. *tomato* DC3000 (NP_793205), 6e^{-49}	Conserved hypothetical protein
PFL_1985	PFL_1985	2213627..2213758	43	4.439	TAAG	None detected	None detected	Hypothetical protein
PFL_1986	PFL_1986	2213809..2214522 (-)	237	26.143	None selected	Repressor C2 from *Salmonella typhimurium* bacteriophage ST64T (NP_720299); 5e^{-21}	Repressor protein C1/C2 family PP3033 from *Pseudomonas putida* KT2440 (AAN68641); 2e^{-64}	Putative LexA-like transcriptional repressor of C1/C2 family (COG1974)
3[b]	*orf3*	2214599..2214871	90	9.740	GAGG	None detected	Putative regulatory protein PP3034 from	Conserved hypothetical

							Pseudomonas putida KT2440 (NP_745178); 4e^{-29}	protein
PFL_1987	PFL_1987	2215205..2215369 (-)	54	5.197	GGGA	None detected	None detected	Hypothetical protein
PFL_1988[c]	PFL_1988	2215220..2215738	172	18.872	AGGA	Hypothetical protein gp14 from *Burkholderia* phage Bcep176 (ABA60015), 5e^{-07}	Hypothetical protein PSPTO3420 from *Pseudomonas syringae* pv. *tomato* DC3000 (NP_793203), 2e^{-85}	Conserved hypothetical phage protein
PFL_1989	PFL_1989	2215930..2218167	746	83.604	GGAG	Hypothetical protein ORF002 from *Staphylococcus* phage 47 (AAX91193), 9e^{-54}	DNA primase domain protein PSPTO3418 from *Pseudomonas syringae* pv. *tomato* DC3000 (NP_793201); 0.0	Putative Ssb-like protein (COG5545)
PFL_1990	PFL_1990	2218181..2218525	114	12.595	GCGAA	None detected	Conserved hypothetical protein PSPTO3417 from *Pseudomonas syringae* pv. *tomato* DC3000 (NP_793200), 9e^{-45}	Conserved hypothetical protein
PFL_1991	*hol*	2219046..2219390	114	11.988	AGGGG	Holin from pyocin locus of *Pseudomonas aeruginosa* (BAA83151); 5e^{-28}	Hypothetical protein Pfl01_1137 from *Pseudomonas fluorescens* Pf0-1 (ABA72880); 2e^{-47}	Holin
PFL_1992	PFL_1992	2219596..2220168	190	20.878	GGAC	None detected	Conserved hypothetical protein PSPTO3413 from *Pseudomonas syringae* pv. *tomato* DC3000 (AAO56891), 2e^{-79}	Conserved hypothetical protein

PFL_1993	PFL_1993	2220134..2222185	683	76.840	CAGAG	Phage terminase gp15 from *Wolbachia* phage WO (BAA89640), $6e^{-109}$	Putative terminase GpA from *Pseudomonas putida* GB-1 (ABY97103), 0.0	Phage terminase (COG5525)
PFL_1994	PFL_1994	2222187..2222393	68	7.601	AAGAG	None detected	Conserved hypothetical protein PSPTO3411 from *Pseudomonas syringae* pv. *tomato* DC3000 (AA056889), $2e^{-23}$	Conserved hypothetical protein
PFL_1995	PFL_1995	2222393..2223874	493	55.288	CAAGG	Phage portal protein from *Wolbachia* sp. bacteriophage WOcauB1(BAD16786), $1e^{-105}$	Phage portal protein, lambda family PP_3044 from *Pseudomonas putida* KT2440 (AE016495), 0.0	Portal protein (COG5511)
PFL_1996	PFL_1996	2223871..2225013	380	40.265	AGGAG	Putative protease Z3097 from *E.coli* O157:H7 prophage CP-933U (AAG57011), $8e^{-31}$	Clp protease PSPTO3409 from *Pseudomonas syringae* pv. *tomato* DC3000 (AAO56887), $7e^{-142}$	Putative ClpP-like protease (COG0740)
PFL_1997	PFL_1997	2225010..2225354	114	11.885	AGGAA	None detected	Conserved hypothetical protein PputGB1_1199 from *Pseudomonas putida* GB-1 (ABY97107), 2e-43	Conserved hypothetical protein
PFL_1998	PFL_1998	2225418..2226413	331	36.382	GGAG	Phage protein gp20 from *Wolbachia* sp. bacteriophage WO (BAA89645), $6e^{-51}$	Conserved hypothetical protein PSPTO3407 from *Pseudomonas syringae* pv. *tomato* DC3000 (AAO56885), $8e^{-156}$	Conserved hypothetical phage protein

PFL_1999	PFL_1999	2226416..2226730	104	11.328	GGAG	None detected	Conserved hypothetical protein PSPTO3406 from *Pseudomonas syringae* pv. *tomato* DC3000 (AAO56884), 4e^{-39}	Conserved hypothetical protein
PFL_2000	PFL_2000	2226727..2227380	217	24.691	GGAG	Putative phage-related protein from phage VP882 of *Vibrio parahaemolyticus* 882 (AAS38501), 1e^{-06}	Conserved hypothetical protein PSPTO3405 from *Pseudomonas syringae* pv. *tomato* DC3000 (AAO56883), 8e^{-75}	Hypothetical phage protein
PFL_2001	PFL_2001	2227373..2227885	170	18.805	GGTG	Hypothetical protein PRF10 from R pyocin region of *P.aeruginosa* PAO1 (BAA83152), 7e^{-28}	Conserved hypothetical protein PSPTO3404 *Pseudomonas syringae* pv. *tomato* DC3000 (AAO56882), 5e^{-66}	Hypothetical phage protein
PFL_2002[d]	PFL_2002	2227882..2228472	196	20.198	GGAG	Putative baseplate assembly protein V from R pyocin region of *P.aeruginosa* PAO1 (BAA83153), 4e^{-53}	Putative tail spike protein gpV from *Pseudomonas entomophila* L48 (AAO56881), 4e^{-67}	Baseplate assembly protein V (COG4540)
PFL_2003	PFL_2003	2228537..2228737	66	7.144	GGAG	None detected	Conserved hypothetical protein PSPTO3402 from *Pseudomonas syringae* pv. *tomato* DC3000 (AAO56880), 3e^{-09}	Conserved hypothetical protein
PFL_2004	PFL_2004	2228742..2229068	108	12.072	GGAG	Putative baseplate assembly protein W from R pyocin region of *P.aeruginosa* PAO1	Putative baseplate assembly protein gpW from *Pseudomonas*	Baseplate assembly protein W (COG3628)

						(BAA83155), 1e^{-35}	*entomophila* L48 (PSEEN4162), 6e^{-43}	
PFL_2005	PFL_2005	2229065..2229946	293	31.192	GGAG	Baseplate J-like protein (baseplate or base of tail fiber) phage phi CTX of *P.aeruginosa* PAO1 (NP_490617), 3e^{-71}	Putative baseplate J-like protein gpJ from *Pseudomonas entomophila* L48 (PSEEN4161), 9e^{-110}	Baseplate J protein (COG3948)
PFL_2006	PFL_2006	2229948..2230553	201	22.268	GGAG	Baseplate assembly protein gpI from Enterobacteria phage P2 (AAD03285), 2e^{-48}	Tail protein I PSPTO3399 from *Pseudomonas syringae* pv. *tomato* DC3000 (AAO56877), 4e^{-77}	Tail protein I (COG4385)
PFL_2007	PFL_2007	2230550..2231776	408	42.749	GGAC	Phage-related tail fibre protein HR2 from R pyocin region of *P.aeruginosa* PAO1 (BAA83158), 5e^{-41}	Putative tail fiber protein H PSPTO3398 from *Pseudomonas syringae* pv. *tomato* DC3000 (AAO56876), 3e^{-72}	Tail fiber protein H (COG5301)
PFL_2008	PFL_2008	2231776..2232558	260	25.745	GGTG	None detected	None detected	Hypothetical protein
PFL_2009	PFL_2009	2232704..2233870	388	42.051	GGAG	Phage tail sheath protein from R pyocin region of *P.aeruginosa* PAO1 (BAA83160), 1e^{-156}	Putative baseplate J-like protein gpFI from *Pseudomonas entomophila* L48 (PSEEN4155), 0.0	Tail sheath protein (COG3497)
PFL_2010	PFL_2010	2233889..2234392	169	18.403	GGAG	Contractile tube protein of *P. aeruginosa* phage PS17 (JC5192), 2e^{-57}	Major tail tube protein PSPTO3395 from *Pseudomonas syringae* pv. *tomato* DC3000 (AAO56873), 8e^{-84}	Major tail tube protein (COG3498)
PFL_2011	PFL_2011	2234404..2234709	101	10.981	AGGAA	Conserved hypothetical protein	Conserved hypothetical protein	Conserved hypothetical

						gp23 from bacteriophage WOcauB1 of *Wolbachia* sp. wCauB (BAD16807), 3e$^{-05\backslash}$	PputW619_4038 from *Pseudomonas putida* W619 (ACA74518), 8e^{-32}	protein
PFL_2012	PFL_2012	2234717..2234821	33	3.964	GGCG	None detected	None detected	Hypothetical protein
PFL_2013	PFL_2013	2234851..2237418	855	88.148	GGTG	Tail tape meausure protein PRF20 from R pyocin region of *P.aeruginosa* PAO1 (BAA83164), 4e^{-47}	Tail tape meausure protein PSPTO3393 from *P. syringae* pv. *tomato* DC3000 (AAO56871), 0.0	Tape measure protein (COG3941)
PFL_2014	PFL_2014	2237427..2238272	281	29.763	GGAG	Protein PRF21 from R pyocin region of *P.aeruginosa* PAO1 (BAA83165), 3e^{-65}	Hypothetical protein PSPTO3392 from *P. syringae* pv. *tomato* DC3000 (AAO56870), 5e^{-115}	Tail formation protein gpU (COG3499)
PFL_2015	PFL_2015	2238247..2238453	68	7.322	GGAG	Phage tail protein PRF22 (GpX) from R pyocin region of *P.aeruginosa* PAO1 (BAA83166), 6e^{-21}	Tail protein X PSPTO3391 from *P. syringae* pv. *tomato* DC3000 (AAO56869), 3e^{-24}	Tail protein X (COG5004)
PFL_2016	PFL_2016	2238450..2238920 (-)	156	16.732	GGAG	None detected	None detected	Hypothetical protein
PFL_2017	PFL_2017	2239001..2240044	347	38.005	GGAG	Phage late control gene D protein PRF23(GpD) from R pyocin region of *P.aeruginosa* PAO1 (BAA83167), 2e^{-96}	Tail protein D PSPTO3390 from *P. syringae* pv. *tomato* DC3000 (AAO56868), 2e^{-160}	Late control protein gpD (COG3500)
PFL_2018	*lys*	2240071..2240622	183	19.846	GGAG	Lytic enzyme PRF24 from R pyocin region of *P.aeruginosa* PAO1 (BAA83168), 1e^{-35}	Putative lysozyme PP_3066 from *Pseudomonas putida* KT2440 (AE016496), 1e^{-82}	Endolysin (COG3179)
PFL_2019	PFL_2019	2240619..2241140	173	18.154	GGTG	Conserved phage protein gp78 from	Hypothetical protein PSPPH_0666 from	Conserved phage protein

Burkholderia cepacia phage Bcep22 (AAQ55012), $4e^{-05}$	*P. syringae* pv. *phaseolicola* 1448a (AAZ35483), $2e^{-42}$

[a] Only expectation values of $1e^{-05}$ and below were considered as significant matches during BLAST database searches; [b] open reading frame(s) not present in the original genome annotation; [d] open reading frame has an alternative start site.
