# Supplementary material for: Mobile genetic elements in the genome of the beneficial rhizobacterium Pseudomonas fluorescens Pf-5
Source: BMC Microbiol. 2009 Jan 13;9:8. doi: 10.1186/1471-2180-9-8 (PMC2647930; doi:10.1186/1471-2180-9-8)
Supplement: Additional file 4 — Sequence analysis of prophage 06 of P. fluorescens Pf-5. Table containing annotation of mobile genetic element prophage 06 in the genome of Pseudomonas fluorescens Pf-5. The following information is provided for each open reading frame: locus tag number, gene name, genome coordinates, length and molecular weight of encoded protein, sequence of putative ribosome binding site, description of the closest GenBank match plus blast E-value, list of functional domains and predicted function. [file 1471-2180-9-8-S4.pdf]

Additional file 4 – Sequence analysis of prophage 06 of *P. fluorescens* Pf-5

CDS	Gene name	Position	Protein length (residues)	Protein mol weight (kDa)	Putative ribosome binding site	Closest protein of phage or pyocin origin (accession no.), blastp E-value [a]	Other similar proteins (accession no.), blastp E-value [a]	Predicted function (functional domains)
PFL_3739 (tRNA-Ser)	*attL*	4338363..4338465 (within tRNA-Ser – PFL_3739)	CGGTCTTGAAAACCGCCGACTGTAACAGGTCCTAGAGTTCGAATCTCTACGCCTCCGCCAAATCTCAACGA AAAAGCCCTGATTTTTCAGGGCTTTTTTGTGT					
PFL_3740[d]	PFL_3740	4339018..4339722	234	26.170	GGAG	Hyp prot STM1053 from Gifsi-2 prophage of *Salmonella typhimurium* LT2 (NP_460027), $1e^{-33}$	Hypothetical protein PFL_3796 from *Pseudomonas fluorescens* Pf-5 (AAY93060), $8e^{-115}$	Phage related conserved protein
PFL_3741	PFL_3741	4339894..4340019	41	4.845	GGAC	None detected	None detected	Hypothetical protein
2[b]	*orf2*	4340357..4341193	278	29.888	CATGG	None detected	Fimbrial protein Rpic12DDRAFT_0572 from *Ralstonia pickettii* 12D (EDN39228), 1e-26	P-pilus assembly protein FimA (COG3539)
PFL_3742	PFL_3742	4341320..4341841 (-)	173	19.114	CAGG	Lysis protein Bcep22gene80 from Burkholderia phage Bcep22 (AAQ55012), 2e-05	Probable phage signal peptide protein PflO1_1173 from *Pseudomonas fluorescens* Pf-01 (ABA72916), $8e^{-53}$	Hypothetical phage protein
PFL_3743	PFL_3743	4342413..4341838 (-)	191	20.768	AGCAG	Hypothetical protein Orf21 from *Pseudomonas aeruginosa* phage D3112 (AAQ94459), $2e^{-29}$	Putative protein P5 from *Pseudomonas putida* KT2440 (AAN67204), $1e^{-66}$	Phage related hypothetical protein
3[b]	*orf3*	4342430..4343254 (-)	274	30.112	ACGG	None detected	Hypothetical protein PputW619_1354 from *Pseudomonas putida* W619 (ACA71859), $4e^{-15}$	Conserved hypothetical protein

4[b]	*orf4*	4343254..4343544 (-)	96	10.410	GTGG	None detected	None detected	Hypothetical protein
PFL_3744	PFL_3744	4346630..4343541 (-)	1029	112.311	GAAGG	Tail tip fiber protein gp62 from *Burkholderia* phage Bcep176 (ABA60063), 0.0	Probable bacteriophage protein Rsc1698 from *Ralstonia solanacearum* GMI1000 (CAD15400), 0.0	Tail tip fiber protein (COG4733)
PFL_3745[d]	PFL_3745	4346688..4347269 (-)	193	20.380	GAGG	Phage protein gp20 from Klebsiella phage phiK02 (AAR83036), $1e^{-41}$	Putative phage tail assembly protein xcc-b100_3327 from *Xanthomonas campestris* pv. *campestris* B100 (CAP52692), 1e-47	Tail sheath protein (COG4723)
5[b]	*orf5*	4347322..4347741 (-)	139	14.391	GAGG	None detected	Putative lipoprotein YPMT1.32 from plasmid pMT1 of *Yersinia pestis* CO92 (NP_395370), $6e^{-13}$	Conserved hypothetical protein
PFL_3746	PFL_3746	4348646..4347840 (-)	268	29.125	GAGG	Hypothetical protein Orf44 of *Shigella flexneri* phage V (NP_599076), $8e^{-27}$	Hypothetical protein UTI89_C5105 from *Escherichia coli* UTI89 (ABE10505), $8e^{-27}$	Phage protein of KilA superfamily
PFL_3747[d]	PFL_3747	4348643..4349617 (-)	324	36.286	GAGG	Putative antirepressor Ant from *Actinobacillus actinomycetemcomitans* phage Aaphi23 (NP_852746), $8e^{-28}$	Putative antirepressor PputGB1_3400 from *Pseudomonas putida* GB-1 (ABY99291), $3e^{-36}$	Putative antirepressor protein (COG3617)
6[b]	*orf6*	4349875..4350303	142	15.833	TGGAA	Probable phage regulatory protein YPO2125 from *Yersinia pestis* CO92 (AD0259), $6e^{-16}$	Putative phage protein y2192 from *Yersinia pestis* KIM (NP_669502), $5e^{-16}$	Conserved hypothetical phage protein
PFL_3748	PFL_3748	4350411..4351133	240	25.710	GGAG	None detected	None detected	Hypothetical protein
7[b]	*orf7*	4351174..4351335 (-)	53	6.023	GAGG	None detected	None detected	Hypothetical

								protein
8[b]	*orf8*	4351350..4351940 (-)	196	22.441	GTGG	None detected	Hypothetical protein VCA0293 from *Vibrio cholerae* O1 biovar eltor str. N16961 (NP_232689), $7e^{-28}$	Conserved hypothetical protein
9[b]	*orf9*	4352112..4352741	209	24.221	GGAG	None detected	Hypothetical protein PCNPT3_06151from *Psychromonas* sp. CNPT3 (EAS39057), 1e-19	Conserved hypothetical protein
PFL_3749	PFL_3749	4352798..4353562 (-)	254	28.637	GGAG	Tail component protein gp19 of *Burkholderia thailandensis* phage phiE125 (NP_536375), $2e^{-62}$	Putative phage protein gp64 from *Burkholderia pseudomallei* 305 (EBA50886), $7e^{-70}$	Tail protein K (COG1310)
PFL_3750	PFL_3750	4353565..4354248 (-)	227	24.958	GAGG	Putative minor tail protein gp18 from *Burkholderia thailandensis* phage phiE125 (NP_536374), $3e^{-52}$	Probable phage hk022 gp18-related protein RSc1694 from *Ralstonia solanacearum* GMI1000 (CAD15396), $2e^{-84}$	Minor phage tail protein (COG4672)
PFL_3751	PFL_3751	4354276..4355469 (-)	397	41.596	CAGG	L-shaped tail fiber protein (LTF) of *E.coli* bacteriophage T5 (AAU05270), $5e^{-43}$	Neck appendage protein precursor GA-1p31 of *Bacillus* phage GA-1 (NP_073695), $5e^{-13}$	Phage tail fiber protein
PFL_3752	PFL_3752	4355466..4355843 (-)	125	14.016	GGCG	Putative tail protein gp16 of Klebsiella bacteriophage phiK02 (AAR83032), $3e^{-28}$	Minor tail protein M PSEEN4176 from *Pseudomonas entomophila* L48 (CAK16867), $9e^{-34}$	Minor phage tail protein M (COG4718)
10[b]	*orf10*	4355861..4356172 (-)	103	11.234	GAGG	None detected	None detected	Hypothetical protein
PFL_3753	PFL_3753	4356172..4359495 (-)	1107	116.614	GAGAG	Putative tail component protein Orf45 from *Pseudomonas*	Phage tail tape measure protein PSPA_2417 from *Pseudomonas*	Putative phage tail length tape measure

						aeruginosa PAS429 phage D3112 (AAQ94483),3e^{-84}	*aeruginosa* PA7 (ABR86240), 0.0	protein (COG5281)
11[b]	*orf11*	4359492..4359743 (-)	83	9.053	GAAGA	Hypothetical protein rtp36 from Enterobacteria phage RTP (CAJ42240), 2e^{-07}	Putative phage protein PSPA_2416 from *Pseudomonas aeruginosa* PA7 (ABR84058), 1e^{-19}	Conserved hypothetical protein
PFL_3754	PFL_3754	4359809..4360189 (-)	125	13.978	GGTG	None detected	Hypothetical protein PSPA7_2415 from *Pseudomonas aeruginosa* PA7 (ABR85104), 6e^{-37}	Conserved hypothetical protein
PFL_3755	PFL_3755	4360193..4360849 (-)	218	22.959	AGGAA	Protein TsbA from Enterobacteria phage TLS (AAR09272), 6e^{-30}	Hypothetical protein Psyr_2783 from *Pseudomonas syringae* pv. *syringae* B728a (AAY37822), 2e^{-48}	Conserved hypothetical phage protein
12[b]	*orf12*	4360938..4361198 (-)	86	9.601	CAGG	None detected	Hypothetical protein	Hypothetical protein
PFL_3756	PFL_3756	4361252..4361668 (-)	138	15.051	AGGA	None detected	Hypothetical protein Psyr_2784 from *Pseudomonas syringae* pv. *syringae* B728a (AAY37823), 1e^{-31}	Conserved hypothetical protein
PFL_3757	PFL_3757	4361665..4362318 (-)	217	23.788	GGTG	Hypothetical protein BPKS7gp02 from *Salmonella* phage KS7 (AAW51205), 4e-14	Conserved hypothetical protein PputGB1_3411 from *Pseudomonas putida* GB-1 (ABY99302), 6e-91	Conserved hypothetical phage protein
PFL_3758	PFL_3758	4362315..4362683 (-)	122	13.560	GGAG	Putative phage protein BB2223 from *Bordetella bronchiseptica* RB50 (NP_888767), 1e^{-12}	Hypothetical protein Psyr_2786 from *Pseudomonas syringae* pv. *syringae* B728a (AAY37825), 1e^{-46}	Conserved hypothetical phage protein
PFL_3759	PFL_3759	4362700..4363203 (-)	167	18.314	GCGAG	Hypothetical protein SPSV3_gp41 from	Hypothetical protein Psyr_2787 from	Conserved hypothetical

						Salmonella phage SETP3 (ABN47370), 5e-09	*Pseudomonas syringae* pv. *syringae* B728a (AYY37826), 5e-70	phage protein
PFL_3760	PFL_3760	4363207..4363515 (-)	102	11.269	GGAG	None detected	Hypothetical protein Psyr_2788 from *Pseudomonas syringae* pv. *syringae* B728a (AYY37827), 6e-14	Conserved hypothetical protein
PFL_3761	PFL_3761	4363567..4364514 (-)	315	33.877	GGAG	Major capsid protein orf18 from *Listonella pelagia* phage phiHSIC (AAW67515), 2e-115	Conserved hypothetical protein PputGB1_3415 from *Pseudomonas putida* GB-1 (ABY99306), 2e-141	Conserved hypothetical protein
PFL_3762	PFL_3762	4364526..4365242 (-)	238	25.772	GGAG	Hypothetical protein ORF009 from *Pseudomonas* phage 73 (YP_001293416), 1e-16	Conserved hypothetical protein PputGB1_3416 from *Pseudomonas putida* GB-1 (ABY99307), 2e-89	Conserved hypothetical phage protein
PFL_3763	PFL_3763	4365352..4365819	155	17.507	AGGAA	None detected	Hypothetical protein BpseB_41036 from *Burkholderia pseudomallei* B7210 (ZP_02477197), 1e-07	Consrved hypothetical protein
PFL_3764	PFL_3764	4365833..4366921 (-)	362	40.052	GGAG	Hypothetical protein BPKS7gp13 from *Salmonella* phage KS7 (AAW51216), $1e^{-56}$	Hypothetical protein Psyr_2729 from *Pseudomonas syringae* pv. *syringae* B728a (AAY37831), $5e^{-148}$	Putative phage minor head protein
PFL_3765	PFL_3765	4366896..4368359 (-)	487	53.748	GGGAT	Phage protein BcepGomrgp05 from *Burkholderia* phage BcepGomr (ABP63576), 4e-93	Hypothetical protein Psyr_2793 from *Pseudomonas syringae* pv. *syringae* B728a (AAY37832), $2e^{-109}$	Conserved hypothetical phage protein
PFL_3766	PFL_3766	4368356..4369657 (-)	433	48.921	AGGA	Putative phage terminase from *Salmonella* phage SETP7 (ABN70692), 4e-93	Hypothetical protein Psyr_2794 from *Pseudomonas syringae* pv. *syringae* B728a	Phage terminase

							(AAY37833), 0.0	
PFL_3767	PFL_3767	4369644..4370105 (-)	153	17.379	GGATG	Hypothetical phage protein y2212 from *Yersinia pestis* KIM (NP_669521), 8e-41	Hypothetical protein Psyr_2795 from *Pseudomonas syringae* pv. *syringae* B728a (AAY37834), 6e-63	Conserved hypothetical phage protein (COG5556)
PFL_3768	PFL_3768	4370137..4370745 (-)	202	22.410	GTGAG	None detected	Hypothetical protein Pput_3382 from *Pseudomonas putida* F1 (ABQ79508), 7e-99	Conserved hypothetical protein
13[b]	*orf13*	4370770..4371120 (-)	115	12.117	GGAAG	None detected	None detected	Hypothetical protein
PFL_3769	PFL_3769	4371186..4371416 (-)	76	8.500	GGAG	Hypothetical protein PHG25p104nc from *Aeromonas* phage 25 (ABF72662), 2e-12	Hypothetical protein Psyr_2800 from *Pseudomonas syringae* pv. *syringae* B728a (AAY37839), 2e-33	Conserved hypothetical phage protein
PFL_3770[d]	*hol*	4371474..4371797 (-)	107	11.502	AGGA	Putative holin from *Pseudomonas* phage F10 (EAZ62533), 5e-24	Phage holin of lambda family PP_1559 from *Pseudomonas putida* KT2440 (AE016345), 3e-34	Holin
14[b]	*orf14*	4371912..4372232 (-)	106	11.728	GGAG	None detected	None detected	Hypothetical protein
PFL_3771[d]	PFL_3771	4372668..4372931 (-)	87	10.109	GAGG	None detected	Hypothetical protein CPS_2864 from *Colwellia psychrerythraea* 34H (AAZ26042); 7e-16	Conserved hypothetical protein
PFL_3772	PFL_3772	4373055..4373750 (-)	231	25.254	GAGG	Phage protein Orf84 *Pseudomonas aeruginosa* phage D3 (AAF80840); 4e-49	Hypothetical protein Pput_4123 from *Pseudomonas putida* F1 (ABQ80247); 3e-66	Conserved hypothetical phage protein
PFL_3773	PFL_3773	4373747..4374337 (-)	196	22.262	GGAG	Protein Orf82 of *Pseudomonas aeruginosa* phage D3 (AAF80838); 1e-39	Hypothetical protein Psyr_2806 from *Pseudomonas syringae* pv. *syringae* B728a	NinG-like conserved phage protein

							(AAY37845), 4e-67	
PFL_3774	PFL_3774	4374337..4374924 (-)	195	22.046	GGAG	Protein Orf79 of *Pseudomonas aeruginosa* phage D3 (NP_061575); 2e-91	Hypothetical prophage protein EcolE2_01003004 from *Escherichia coli* E22 (ZP_00728702); 6e-36	Conserved hypothetical phage protein
PFL_3775	PFL_3775	4374917..4375717 (-)	266	28.401	GGAG	Hypothetical protein F116p33 from *Pseudomonas* phage F116 (AAT47229), 6e-41	Hypothetical protein Psyr_2809 from *Pseudomonas syringae* pv. *syringae* B728a (AAY37848), 3e-74	Conserved hypothetical phage protein
PFL_3776	PFL_3776	4375704..4376678 (-)	324	36.041	GGAG	Hypothetical protein F116p32 from *Pseudomonas* phage F116 (AAT47228), 6e-37	Hypothetical protein Psyr_2810 from *Pseudomonas syringae* pv. *syringae* B728a (AAY37849), 4e-162	Conserved hypothetical phage protein
15[b]	*orf15*	4376680..4377099 (-)	139	15.333	GGTG	Protein gp75 from *Mycobacterium* phage Tweety (ABQ86144), 4e-07	Putative helicase sce8204 from *Sorangium cellulosum* 'So ce 56' (CAN98374), 1e-08	Putative endonuclease (COG1403)
PFL_3777	PFL_3777	4377096..4378115 (-)	339	38.379	CAGG	Putative DNA-binding protein (Roi) from *Pseudomonas* phage F10 (YP_001293393), 3e-41	Putative prophage antirepressor from *Pseudomonas putida* GB-1 (ABY99331), 1e-67	Putative DNA-binding Roi protein (COG3646)
PFL_3778	PFL_3778	4378344..4378748 (-)	134	14.626	GAAGG	None detected	None detected	Hypothetical protein
PFL_3779[d]	PFL_3779	4372668..4372931 (-)	191	20.984	AGAAG	None detected	Hypothetical protein Psyr_2811 from *Pseudomonas syringae* pv. *syringae* B728a (AAY37850), 5e-40	Conserved hypothetical protein
PFL_6232	PFL_6232	4379256..4379459 (-)	67	7.173	GTGG	Cro-like protein from *Pseudomonas* phage F116 (AAT45880), 2e-	Hypothetical protein EcolO15_30369 from *Escherichia coli*	Cro-like phage repressor

						06	O157:H7 str. EC4501 (ZP_0279077), 2e-07	
PFL_3780	*cro*/CI	4380364..4379576	262	29.061	AATAA	Transcriptional regulator F116p29 from *Pseudomonas* phage F116 (AAT45979), 5e-48	Transcriptional regulator PFL_2126 of Cro/CI family from *Pseudomonas fluorescens* Pf-5 (AAY91400), 2e-80	Cro/C1-type repressor protein (COG2932)
PFL_3781[d]	PFL_3781	4380420..4380965	181	19.221	GGAC	None detected	Hypothetical protein PTD2_04771 from *Pseudoalteromonas tunicata* D2 (EAR26471), 3e-08	Conserved hypothetical protein
16[b]	*orf16*	4381010..4381246	78	8.746	GGAG	None detected	Conserved hypothetical protein PP3898 from *Pseudomonas putida* KT2440 (AE016583); 1e-21	Conserved hypothetical protein
17[b]	*orf17*	4381398..4382315	305	34.953	AAGGA	None detected	Hypothetical protein Pden_2054 from *Paracoccus denitrificans* PD1222 (ABL70146), 9e-62	Conserved hypothetical protein
18[b]	*orf18*	4382440..4383309	289	31.072	GGAG	Hypothetical protein SPSV3_gp50 from *Salmonella* phage SETP3 (ABN47379), 9e-06	Hypothetical protein Pput_3368 from *Pseudomonas putida* F1 (ABQ79494); 6e-14	Conserved hypothetical phage protein
19[b]	*orf19*	4383868..4384068	66	7.096	GGAG	None detected	None detected	Hypothetical protein
PFL_3782[d]	PFL_3782	4384141..4384341	66	7.230	GAGG	None detected	Hypothetical protein Pput_4133 from *Pseudomonas putida* F1 (ABQ80257), 2e-25	Conserved hypothetical protein
PFL_3783	PFL_3783	4384849..4385280	143	16.040	GGAG	None detected	None detected	Hypothetical protein
PFL_3784	PFL_3784	4385268..4385468	66	7.368	AGAG	None detected	Hypothetical protein	Conserved

							Psyr_2819 from *P. syringae* pv. *syringae* B728a (AAY37858), 4e-12	hypothetical protein
PFL_3785	PFL_3785	4385465..4385665	66	7.667	GGAG	None detected	None detected	Hypothetical protein
PFL_3786	PFL_3786	4385662..4385835	57	6.075	GGAG	None detected	None detected	Hypothetical protein
PFL_3787	PFL_3787	4385975..4386721	248	27.801	GGAG	Hypothetical protein F116p19 from *Pseudomonas* phage F116 (AAT47218), 5e-40	Hypothetical protein Pput_3410 from *Pseudomonas putida* F1 (ABQ79536), 2e-42	Conserved hypothetical phage protein
PFL_3788[d]	PFL_3788	4386718..4387281	187	21.203	GGTG	Phage protein Orf027 from *Staphylococcus* phage 42E (AAX91139); 2e-11	Hypothetical protein Pput_3411 from *Pseudomonas putida* F1 (ABQ79537), 2e-74	Conserved hypothetical phage protein
PFL_3789	PFL_3789	4387289..4387516	75	8.138	GAGG	None detected	None detected	Hypothetical protein
20[b]	*orf20*	4387513..4387974	153	17.159	GGAG	None detected	Hypothetical proteins PP_3907 from *Pseudomonas putida* KT2440 (AAN69501), 2e-51	Conserved hypothetical protein
PFL_3790	PFL_3790	4388056..4388367	103	11.534	GAGG	None detected	None detected	Hypothetical protein
21[b]	*orf21*	4388364..4387974 (-)	100	11.124	GGAG	None detected	None detected	Hypothetical protein
22[b]	*orf22*	4388758..4389312 (-)	184	20.420	GGAG	None detected	None detected	Hypothetical protein
23[b]	*orf23*	4389570..4389312	72	7.996	GAAGG	None detected	None detected	Hypothetical protein
PFL_3791	PFL_3791	4390090..4390848	252	29.117	GGAG	None detected	Hypothetical protein pCAR1_p090 from plasmid pCAR1 of *Pseudomonas resinovorans*	Conserved hypothetical protein

							(BAC41609); 2e-49	
24[b]	*orf24*	4390913..4391575 (-)	220	24.712	GGTG	None detected	Hypothetical protein V12G01_17162 from *Vibrio alginolyticus* 12G01 (EAS76997), 6e-12	Conserved hypothetical protein
PFL_3792	PFL_3792	4391626..4393281	551	58.551	GGAG	Dam DNA methyltransferase from Enterobacteria phage P7 (AAQ07538), 3e-81	DNA-cytosine methyltransferase PP_3912 from *Pseudomonas putida* KT2440 (AE016584), 0.0	Putative Cytosine-C5-specific DNA methylase (COG0270)
PFL_3793	PFL_3793	4393336..4393491	51	5.958	GGAG	None detected	None detected	Hypothetical protein
25[b]	*orf25*	4393528..4393770	80	9.206	GGAG	Hypothetical protein PPF10_gp029 from *Pseudomonas* phage F10 (YP_001293373), 2e-07	Hypothetical protein PSEEN2227 from *Pseudomonas entomophila* L48 (CAK15046), 1e-23	Conserved hypothetical phage protein
PFL_3794	*int7*	4393772..4394821	349	40.200	GGTG	Integrase PPF10_gp028 from *Pseudomonas* phage F10 (YP_001293372), 1e-100	Site-specific recombinase PSSEEN2226 from *Pseudomonas entomophila* L48 (CAK15045), 6e-163	Putative phage integrase
	attR	4394946..4395048	CGGTCTTGAAAACCGCCGACTGTAACAGGTCCTAGAGTTCGAATCTCTACGCCTCCGCCAAATCTCAACGA GAAAGCCCTGATTTTTCAGGGCTTTTTTGTGT					
PFL_3795	PFL_3795	4395264..4395443 (-)	na	na	na			Degenerate IS5 family transposase
PFL_3796	PFL_3796	4396430..4397113	227	25.373	GAGG	Hypothetical protein F116p67 from *Pseudomonas* phage F116 (AAT47256), 2e-81	Hypothetical protein PFL_3740 from *Pseudomonas fluorescens* Pf-5 (AAY93005), 2e-114	Conserved hypothetical protein (COG2135)
26[b]	*orf26*	4397357..4397668	103	11.628	GGAG	None detected	Hypothetical protein Bpse38_13628 from	Conserved hypothetical

							Burkholderia thailandensis MSMB43 (ZP_02464405), 1e-09	protein
PFL_3797	PFL_3797	4397801..4398301 (-)	166	18.209	GGTG	Phage tail protein from *Salmonella* phage E1 (CAM33114), 5e-06	Conserved hypothetical protein from *Pseudomonas putida* W619 (ACA71862), 2e-14	Conserved hypothetical phage protein
PFL_3798	*lys*	4398298..4398837 (-)	179	19.273	GCGG	Lytic protein from *Pseudomonas* phage F10 (YP_001293405), 2e-44	Predicted chitinase from *Pseudomonas aeruginosa* 2192 (ZP_00973864), 5e-51	Endolysin (COG3179)
PFL_3799	PFL_3799	4398828..4398923	31	3.462	None detected	None detected	None detected	Hypothetical protein
PFL_3780	PFL_3780	4399227..4400075	282	30.161	AGGA	Hypothetical protein PaP3p20 from *Pseudomonas aeruginosa* phage PaP3 (AAL85552), 1e-22	Phage particle protein from *Pseudomonas* phage LUZ24 (CAP45450), 5e-21	Conserved hypothetical phage protein

```
Alignment   attL CGGTCTTGAAAACCGCCGACTGTAACAGGTCCTAGAGTTCGAATCTCTACGCCTCCGCCAAATCTCAACGAAAAAGCCCTGATTTTTCAGGGCTTTTTTGTGT
of attL          ::::::::::::::::::::::::::::::::::::::::::::::::::::::::::::::::::::::: :::::::::::::::::::::::::::::::
and attR    attR CGGTCTTGAAAACCGCCGACTGTAACAGGTCCTAGAGTTCGAATCTCTACGCCTCCGCCAAATCTCAACGAGAAAGCCCTGATTTTTCAGGGCTTTTTTGTGT
```

[a] Only expectation values of $1e^{-05}$ and below were considered as significant matches during BLAST database searches; [b] open reading frame(s) not present in the original genome annotation; [c] open reading frame contains internal frameshifts; [d] open reading frame has an alternative start site.
