# Supplementary material for: Mobile genetic elements in the genome of the beneficial rhizobacterium Pseudomonas fluorescens Pf-5
Source: BMC Microbiol. 2009 Jan 13;9:8. doi: 10.1186/1471-2180-9-8 (PMC2647930; doi:10.1186/1471-2180-9-8)
Supplement: Additional file 5 — Sequence analysis of putative integrase genes from P. fluorescens Pf-5. Table containing annotation of putative integrase genes present in the genome of Pseudomonas fluorescens Pf-5. The following information is provided for each open reading frame: locus tag number, gene name, genome coordinates, length and molecular weight of encoded protein, sequence of putative ribosome binding site, description of the closest GenBank match plus blast E-value, list of functional domains and predicted function. [file 1471-2180-9-8-S5.pdf]

Additional file 5 – Sequence analysis of putative integrase genes from *P. fluorescens* Pf-5

CDS	Gene name	Position	Protein length (residues)	Protein mol weight (kDa)	Putative ribosome binding site	Closest protein of phage or pyocin origin (accession no.), blastp E-value [a]	Other similar proteins (accession no.), blastp E-value [a]	Predicted function (functional domains)
PFL_1101	*xerD*	1256663..1257559	298	33.856	GAGT	None detected	site-specific recombinase XerD from *Pseudomonas fluorescens* Pf0-1 (ABA72768), 8e-162	Integrase/recombinase XerD (COG4973)
PFL_1843[c]	*int1*	2044797..2045261(-)	154	17.618	GGAGG	Phage integrase from *Pseudomonas aeruginosa* phage phiCTX (NP_490644); 1e-115	Putative transposase IntR from putative *E.coli* lambdoid prophage Rac (P76056); 1e-31	Defective phage integrase associated with prophage 02 (COGs 4974, 0582)
PFL_1976	*int2*	2207060..2208202	380	43.927	GTAG	Shufflon-specific recombinase from plasmid ColIb-P9 (AAZ05392), 4e-21	Phage integrase family protein PputGB1_1711 from *Pseudomonas putida* GB-1 (YP_001667950), 0.0	Putative phage integrase associated with prophage 03
int3[b]	*int3*	2338455..2339156 (-)	233	26.312	GTGG	Phage integrase from *Shigella flexneri* phage SfX (AAD10295); 2e-06	Putative integrase PputGB1_3473 from *Pseudomonas putida* GB-1 (ABY99364), $8e^{-99}$	P22-like phage integrase associated with prophage 04
PFL_3009	*int4*	3461943..3463166	407	46.340	GTGA	Putative prophage integrase PSPPH_2793 from *Pseudomonas syringae* pv.	Putative site-specific recombinase from *Magnetospirillum gryphiswaldense* MSR-1 (CAM76684), 1e-114	Putative phage integrase (COG4974)

						phaseolicola 1448A (AAZ33587), 2e-103		
PFL_3010	*int5*	3463166..3464710	514	57.496	GGATG	None detected	Putative site-specific recombinase from *Magnetospirillum gryphiswaldense* MSR-1 (CAM76079), 5e-103	Putative integrase (COG4974)
PFL_3042	*int6*	3507541..3508482 (-)	313	35.473	GGAG	Multiple genome hits	Putative integrase Pf0l_2840 from *Pseudomonas fluorescens* Pf0-1 (ABA74581), 1e-142	Putative phage integrase (COG4973)
PFL_3794	*int7*	4393772..4394821	349	40.200	GGTG	Integrase PPF10_gp028 from *Pseudomonas* phage F10 (YP_001293372), 1e-100	Site-specific recombinase PSSEEN2226 from *Pseudomonas entomophila* L48 (CAK15045), 6e-163	Putative lambda-phage integrase associated with prophage 06.
PFL_4752	*int8*	5491834..5493171	445	50.922	GAGG	Multiple genome hits	Putative integrase PA2G_05424 from *Pseudomonas aeruginosa* 2192 (EAZ62001), 3e-124	Putative integrase associated with mobile island PFGI-1 (COG4973)
PFL_4977	*int9*	5728657..5729898	413	46.144	GGAT	Site-specific recombinase from PAI-R1 island of *Pseudomonas viridiflava* ME3.1b (AAT96071), 0.0	Putative site-specific recombinase from *Acinetobacter baumannii* AYE (CAM87726), 4e-80	Putative P4-like phage integrase associated with prophage 07 (COG0582)
PFL_6015	*xerC (sss)*	6834858..6835754	298	33.523	GGTAC	Multiple genome hits	Site-specific recombinase Sss/XerC from	Site-specific recombinase Sss/XerC (COG4973)

Pseudomonas fluorescens F113 (AA017715), 4e-150

[a] Only expectation values of $1e^{-05}$ and below were considered as significant matches during BLAST database searches; [b] open reading frame(s) not present in the original genome annotation; [c] open reading frame contains internal frameshifts; [d] open reading frame has an alternative start site.
