# Supplementary material for: Mobile genetic elements in the genome of the beneficial rhizobacterium Pseudomonas fluorescens Pf-5
Source: BMC Microbiol. 2009 Jan 13;9:8. doi: 10.1186/1471-2180-9-8 (PMC2647930; doi:10.1186/1471-2180-9-8)
Supplement: Additional file 6 — Sequence analysis of prophage 02 of P. fluorescens Pf-5. Table containing annotation of mobile genetic element prophage 02 in the genome of Pseudomonas fluorescens Pf-5. The following information is provided for each open reading frame: locus tag number, gene name, genome coordinates, length and molecular weight of encoded protein, sequence of putative ribosome binding site, description of the closest GenBank match plus blast E-value, list of functional domains and predicted function. [file 1471-2180-9-8-S6.pdf]

Additional file 6 – Sequence analysis of prophage 02 of *P. fluorescens* Pf-5

CDS	Gene name	Position	Protein length (residues)	Protein mol weight (kDa)	Putative ribosome binding site	Closest protein of phage or pyocin origin (accession no.), blastp E-value[a]	Other similar proteins (accession no.), organism, blastx E-value[a]	Predicted function (functional domains)
	attL	2042157..2042216	TCGAATCTCTCCTTCACCGCCACATTCTACAACCACAAACCCCTGACTTTCCTAAAGAAA					
PFL_1842	PFL_1842	2042784..2044547	610	68.526	GGCG	Hypothetical protein VIBHAR_05008 from *Streptococcus thermophilus* integrative conjugative element ICESt1(ABU72915); 1e-13	Hypothetical protein VIBHAR_05008 from *Vibrio harveyi* ATCC BAA-1116 (ABU72915); 6e-78	Putative mobile element-related protein (COG3593)
PFL_1843[c]	*int1*	2044797..2045261(-)	154	17.618	GGAGG	Phage integrase from *Pseudomonas aeruginosa* phage phiCTX (NP_490644); 1e-115	Putative transposase IntR from putative *E.coli* lambdoid prophage Rac (P76056); 1e-31	Putative phage integrase (COGs 4974, 0582)
PFL_1844	PFL_1844	2046023..2046211(-)	62	7.084	None detected	None detected	None detected	Hypothetical protein
PFL_1845	PFL_1845	2046050..2047222	390	45.502	GCTGG	None detected	Gp5 domain protein PSPA7_5076 from *Pseudomonas aeruginosa* PA7 (ABR83565); 2e-96	Conserved hypothetical protein
PFL_1846	PFL_1846	2047411..2049387(-)	658	75.366	GGAAG	None detected	Hypothetical protein Shewmr4_0837 from *Shewanella* sp. MR-4 (ABI37917); 2e-155	Conserved hypothetical protein (COG 3593)
	attR	2050454..2050513	TCGAATCTCTCCTTCACCGCCACATTCTACAAACACAAACCCCTGACTTTCCTAAAGAAA					
Alignment of *attL* and *attR:*		`attL TCGAATCTCTCCTTCACCGCCACATTCTACAACCACAAACCCCTGACTTTCCTAAAGAAA`<br>`     :::::::::::::::::::::::::::::::: :::::::::::::::::::::::::::`<br>`attR TCGAATCTCTCCTTCACCGCCACATTCTACAAACACAAACCCCTGACTTTCCTAAAGAAA`						

[a] Only expectation values of $1e^{-05}$ and below were considered as significant matches during BLAST database searches; [c] part of a longer open reading frame with internal frameshifts.
