# Supplementary material for: Mobile genetic elements in the genome of the beneficial rhizobacterium Pseudomonas fluorescens Pf-5
Source: BMC Microbiol. 2009 Jan 13;9:8. doi: 10.1186/1471-2180-9-8 (PMC2647930; doi:10.1186/1471-2180-9-8)
Supplement: Additional file 7 — Sequence analysis of prophage 04 of P. fluorescens Pf-5. Table containing annotation of mobile genetic element prophage 04 in the genome of Pseudomonas fluorescens Pf-5. The following information is provided for each open reading frame: locus tag number, gene name, genome coordinates, length and molecular weight of encoded protein, sequence of putative ribosome binding site, description of the closest GenBank match plus blast E-value, list of functional domains and predicted function. [file 1471-2180-9-8-S7.pdf]

Additional file 7 – Sequence analysis of prophage 04 of *P. fluorescens* Pf-5

CDS	Gene name	Position	Protein length (residues)	Protein mol weight (kDa)	Putative ribosome binding site	Closest protein of phage or plasmid origin (accession no.), blastp E-value [a]	Other similar proteins (accession no.), blastP E-value [a]	Predicted function (functional domains)
PFL_2119	tRNA-Pro *(attL)*	2338296..2338372	CGGGGCGTAGCGCAGTCCGGTAGCGCACTAGCATGGGGTGCTAGGGGTCGAGTGTTCGAATCACTCCGTCC CGACCA					
1[b]	*int3*	2338455..2339156 (-)	233	26.312	GTGG	Phage integrase from *Shigella flexneri* phage SfX (AAD10295); $2e^{-06}$	Putative integrase PputGB1_3473 from *Pseudomonas putida* GB-1 (ABY99364), $8e^{-99}$	Phage integrase
2[b]	*orf2*	2339231..2339893	220	24.660	GGCG	Putative replication protein SfVp39 from *Shigella flexneri* bacteriophage V (NP_599071); $4e^{-05}$	None detected	Putative phage related conserved protein
PFL_2120	PFL_2120	2339491..2339670	59	6.438	GTGAA	None detected	Hypothetical protein PFL_3776 from *Pseudomonas fluorescens* Pf-5 (AAY93041), 7e-22	Conserved hypothetical protein
PFL_2121	PFL_2121	2339919..2340170	83	9.154	GTGAG	Phage protein Orf84 from *Pseudomonas aeruginosa* phage D3 (NP_061580); $1e^{-09}$	Hypothetical protein PFL_3772 from *Pseudomonas fluorescens* Pf-5 (AAY93037), 6e-36	Conserved phage related protein
3[bc]	*orf3*	2341690..2342074	77	8.460	GAGG	L-shaped tail fiber protein of *E.coli* bacteriophage T5 (P13390), $7e^{-12}$	None detected	Tail fiber protein
4[b]	*orf4*	2342156..2343202	348	38.797	AGGA	None detected	Putative acyltransferase PTD2_11199 from *Pseudoalteromonas tunicata* D2 (EAR29378); $6e^{-19}$	Putative acyltransferase (COG1835)
PFL_2122[d]	PFL_2122	2342156..2344091	283	31.778	TAAG	Putative minor phage tail protein L from *Burkholderia pseudomallei* phage	Hypothetical protein PFL_3749 from *Pseudomonas fluorescens* Pf-5 (AAY93014), 2e-48	Putative minor tail protein similar to lambda protein L

						phi1026b (NP_945047); $5e^{-29}$		(COG4672)
PFL_2123	PFL_2123	2344115..2344513	132	15.082	CAAG	Tail component protein gp19 of *Burkholderia thailandensis* phage phiE125 (NP_536375), $5e^{-19}$	Hypothetical protein PFL_3749 from *Pseudomonas fluorescens* Pf-5 (AAY93014), 4e-57	Conserved phage related protein
5[b]	*orf5*	2344589..2345038	149	16.397	AGGAA	None detected	None detected	Hypothetical protein
6[b]	*orf6*	2345493..2346008	171	18.287	GGGCG	Putative protein gp80 from *Burkholderia* phage Bcep22 (AAQ55012); 6e-06	Hypothetical protein PFL_2019 from *Pseudomonas fluorescens* Pf-5 (AAY91305), 3e-31	Putative phage protein
7[b]	*orf7*	2346162..2346701 (-)	179	18.591	GAGG	None detected	Conserved hypothetical protein XCC3128 from *Xanthomonas campestris* pv. *campestris* ATCC 33913 (NP_638475), $1e^{-26}$	Conserved hypothetical protein
PFL_2124	PFL_2124	2347171..2347848 (-)	225	25.124	AGGGG	None detected	Hypothetical protein PFL_3740 from *Pseudomonas fluorescens* Pf-5 (AAY93005), 5e-108	Conserved hypothetical protein (COG2135)
8[b]	*orf8*	2347920..2348480 (-)	186	21.430	GAGG	None detected	Putative ATP-dependent DNA ligase PflO1_2097 from *Pseudomonas fluorescens* PfO-1 (ABA73840); $3e^{-61}$	Putative ATP-dependent DNA ligase
9[bc]	*orf9*	2348722..2348895	57	6.244	na	None detected	Hypothetical protein PFL_2878 from *Pseudomonas fluorescens* Pf-5 (AAY92150), 4e-06	Putative cell wall glycosyltransferase (COG0463)
PFL_2125	PFL_2125	2349053..2349262 (-)	69	7.647	GAGG	None detected	Hypothetical protein	Hypothetical protein
PFL_2126	PFL_2126	2349714..2350442 (-)	242	27.075	CTGG	Pyocin regulator PrtR from *Pseudomonas aeruginosa* (Q06553); $3e^{-50}$	Hypothetical protein PFL_3780 from *Pseudomonas fluorescens* Pf-5 (AAY93045), 3e-80	Cro/C1-type repressor protein (COG2932)

10[b]	*orf10*	2350540..2350722	60	6.480	GGCAG	None detected	Hypothetical protein PFL_3780 from *Pseudomonas fluorescens* Pf-5 (AAY93045), 3e-80	Conserved hypothetical protein
PFL_2127	*llpA1*	2350955..2351797	280	31.104	AGGT	None detected	Putidacin PFL_1229 from *Pseudomonas fluorescens* Pf-5 (AAY90516), 2e-155	Bacteriocin putidacin L1

[a] Only expectation values of $1e^{-05}$ and below were considered as significant matches during BLAST database searches; [b] open reading frame(s) not present in the original genome annotation; [c] part of a longer open reading frame with internal frameshifts; [d] open reading frame has an alternative start site.
