# Supplementary material for: Mobile genetic elements in the genome of the beneficial rhizobacterium Pseudomonas fluorescens Pf-5
Source: BMC Microbiol. 2009 Jan 13;9:8. doi: 10.1186/1471-2180-9-8 (PMC2647930; doi:10.1186/1471-2180-9-8)
Supplement: Additional file 8 — Sequence analysis of prophage 05 of P. fluorescens Pf-5. Table containing annotation of mobile genetic element prophage 05 in the genome of Pseudomonas fluorescens Pf-5. The following information is provided for each open reading frame: locus tag number, gene name, genome coordinates, length and molecular weight of encoded protein, sequence of putative ribosome binding site, description of the closest GenBank match plus blast E-value, list of functional domains and predicted function. [file 1471-2180-9-8-S8.pdf]

Additional file 8 – Sequence analysis of prophage 05 of *P. fluorescens* Pf-5

CDS	Gene name	Position	Protein length (residues)	Protein mol weight (kDa)	Putative ribosome binding site	Closest protein of phage or pyocin origin (accession no.), blastp E-value [a]	Other similar proteins (accession no.), blastP E-value [a]	Predicted function (functional domains)
	attL	3979487..3979570	GCAATAAAAAACCCCGTAGACGTTAATCTACGGGGTTTTCAAGGGTGGAGGCCGAGGTCGGAATCGAACCGGCGTAGGCGGATT					
PFL_3464[d]	PFL_3464	3979525..3980079 (-)	184	20.973	GGTG	Host specificity protein J from *Shewanella oneidensis* MR-1 prophage LambdaSo (NP_718509); $3e^{-56}$	Putative phage tail component protein Paer2_01001065 from *Pseudomonas aeruginosa* 2192 (ZP_00975650); $2e^{-63}$	Phage tail protein J (COG4733)
1[b]	*orf1*	3980099..3980626 (-)	175	18.426	GGAATG	None detected	Putative flavoprotein HCH_01405 from *Hahella chejuensis* KCTC 2396 (ABC28268); $1e^{-35}$	Putative NADPH-dependent FMN reductase (COG0431)
2[bc]	*int*	3981099..3981948	281	32.077	GTGAG	Multiple prophage genome hits	Site-specific recombinase PSEEN2226 from *Pseudomonas entomophila* L48 (CAK15045); $5e^{-100}$	Putative inactive phage integrase
	attR	3982003..3982086 (within tRNA-Cys – PFL_3456)	GCAATAAAAAACCCCGTAGACGTTAATCTACGGGGTTTTCAAGGGTGGAGGCCGAGGTCGGAATCGAACCGGCGTAGGCGGATT					

Alignment of *attL* and *attR:*

```
attL GCAATAAAAAACCCCGTAGACGTTAATCTACGGGGTTTTCAAGGGTGGAGGCCGAGGTCGGAATCGAACCGGCGTAGGCGGATT
     ::::::::::::::::::::::::::::::::::::::::::::::::::::::::::::::::::::::::::::::::::::
attR GCAATAAAAAACCCCGTAGACGTTAATCTACGGGGTTTTCAAGGGTGGAGGCCGAGGTCGGAATCGAACCGGCGTAGGCGGATT
```

[a] Only expectation values of $1e^{-05}$ and below were considered as significant matches during BLAST database searches; [b] open reading frame(s) not present in the original genome annotation; [c] open reading frame contains internal frameshifts; [d] open reading frame has an alternative start site.
