# Supplementary material for: Mobile genetic elements in the genome of the beneficial rhizobacterium Pseudomonas fluorescens Pf-5
Source: BMC Microbiol. 2009 Jan 13;9:8. doi: 10.1186/1471-2180-9-8 (PMC2647930; doi:10.1186/1471-2180-9-8)
Supplement: Additional file 9 — Sequence analysis of island 01 of P. fluorescens Pf-5. Table containing annotation of mobile genetic element island 01 in the genome of Pseudomonas fluorescens Pf-5. The following information is provided for each open reading frame: locus tag number, gene name, genome coordinates, length and molecular weight of encoded protein, sequence of putative ribosome binding site, description of the closest GenBank match plus blast E-value, list of functional domains and predicted function. [file 1471-2180-9-8-S9.pdf]

Additional file 9 – Sequence analysis of island 01 of *P. fluorescens* Pf-5

CDS	Gene name	Position	Protein length (residues)	Protein mol weight (kDa)	Putative ribosome binding site	Closest protein of phage or pyocin origin (accession no.), blastp E-value [a]	Other similar proteins (accession no.), blastp E-value [a]	Predicted function (functional domains)
PFL_4658 (pseudo-tRNA)	*attL*	5378468..5378541 (-)	CCCTTCCTAGAGCAGTTCAGTCCAATTGTCTTTTAACCAATTGGTCGTAGGTTCGAATCCCACACGACCCACCA					
1[b]	*soj*	5378616.. 5379488	290	31.681	GAGG	Putative Soj protein from *Pseudomonas syringae pv. phaseolicola* 1302A (CAI36042), 3e^{-105}	Chromosome partitioning related protein PA14_58910 from *Pseudomonas aeruginosa* UCBPP-PA14 (ABJ13810), 6e^{-105}	Partitioning protein (COGs1192, 0455, 0489)
PFL_4659	PFL_4659	5379490..5380242	250	28.156	GGAAG	None detected	Hypothetical protein Psyr_1533 from *Pseudomonas syringae pv. syringae* B728a (AAY36583), 7e^{-68}	Conserved hypothetical protein
PFL_4660	PFL_4660	5380239..5380622	127	14.148	GGTG	None detected	Hypothetical protein CtesDRAFT_3559 from *Comamonas testosteroni* KF-1 (EAV12559), 2e^{-11}	Conserved hypothetical protein
2[b]	*orf2*	5380619..5380903	94	10.016	GGAGG	None detected	None detected	Hypothetical protein
3[b]	*orf3*	5380900..5381181	93	10.185	GGAG	None detected	Hypothetical protein Pput_4138 from *Pseudomonas putida* F1 (ABQ80262), 5e^{-24}	Conserved hypothetical protein
PFL_4661	PFL_4661	538118..5381870	229	25.435	GGAGG	None detected	Hypothetical protein PA14_58960 from *Pseudomonas aeruginosa* UCBPP-PA14 (ABJ13815), 4e^{-65}	Conserved hypothetical protein

PFL_4662	PFL_4662	5381933..5382538	201	22.857	GAGG	Gp46 from *Burkholderia* phage phi1026b (AAR23197), 2e-13	Hypothetical protein RSc1656 from *Ralstonia solanacearum* GMI1000 (CAD15358), 1e-15	Conserved hypothetical protein
PFL_4663	*dnaB*	5382535..5383884	449	49.306	GGAGG	None detected	Putative replicative DNA helicase from *Pseudomonas aeruginosa* (ABR13456), 2e-173	Putative replicative DNA helicase (COG0305)
4[b]	*orf4*	5383881..5384069	62	7.266	GGAGG	None detected	Hypothetical protein from *Pseudomonas aeruginosa* (ABR13455), 4e-05	Conserved hypothetical protein
PFL_4664	PFL_4664	5384059..5384577	172	19.060	GGAGG	None detected	Hypothetical protein PA2G_05504 from *Pseudomonas aeruginosa* 2192 (EAZ62078), 4e-41	Conserved hypothetical protein
5[b]	*orf5*	5384570..5384800	76	8.613	AGGA	None detected	Hypothetical protein XCV2294 from *Xanthomonas campestris pv. vesicatoria* 85-10 (CAJ23971), 2e-10	Conserved hypothetical protein
PFL_4665[d]	PFL_4665	5384800..5385801	333	37.513	AGGA	None detected	Nucleoid-associated protein NdpA from *Pseudomonas fluorescens* Pf0-1 (ABA72726), 1e-172	Putative nucleoid-associated protein (COG3081)
6[b]	*orf6*	5385819..5386145	108	12.022	GGAG	None detected	Arc domain-containing protein Pput_1445 from *Pseudomonas putida* F1 (ABQ77603), 1e-11	Conserved hypothetical protein
PFL_4666	PFL_4666	5386147..5387820	557	61.608	GGTG	None detected	Hypothetical protein from *Pseudomonas*	Conserved hypothetical

							aeruginosa (AAP22516), 1e-179	protein
PFL_4667	PFL_4667	5387862..5388488	208	23.632	GGAGG	None detected	Hypothetical protein from *Pseudomonas aeruginosa* 2129 (ABR13446), 1e-53	Conserved hypothetical protein
PFL_4668	PFL_4668	5388485..5389765	426	46.771	GGCG	None detected	Hypothetical protein PA14_59100 from *Pseudomonas aeruginosa* UCBPP-PA14 (ABJ13827), 3e-130	Conserved hypothetical protein
PFL_4669	PFL_4669	5390321..5391052	243	27.346	AGGA	None detected	Hypothetical protein PA14_59130 from *Pseudomonas aeruginosa* UCBPP-PA14 (ABJ13830), 7e-89	Conserved hypothetical protein
PFL_4670	PFL_4670	5391049..5391594	181	19.825	GGTG	None detected	Hypothetical protein PACG_04945 from *Pseudomonas aeruginosa* C3719 (ABR13439), 7e-62	Conserved hypothetical protein
PFL_4671	PFL_4671	5391609..5392115	168	18.863	GGAG	None detected	Putative ssb protein PACG_04944 from *Pseudomonas aeruginosa* C3719 (ABR13439), 3e-56	Putative single-stranded DNA binding protein (COG0629)
7[b]	*orf7*	5392794..5392991	65	7.240	GAGG	None detected	Hypothetical protein PA14_59160 from *Pseudomonas aeruginosa* UCBPP-PA14 (ABJ13833), 7e-89	Conserved hypothetical protein
PFL_4672	*topA*	5393086..5395002	638	71.255	GGAC	None detected	Putative topoisomerase I PA2G_05490 from *Pseudomonas*	Putative topoisomerase IA (COG0550)

							aeruginosa 2192 (EAZ62064), 0.0	
PFL_4673	PFL_4673	5395092..5396972	626	70.597	GGAG	None detected	Hypothetical protein PA14_59200 from *Pseudomonas aeruginosa* UCBPP-PA14 (ABJ13837), 1e-124	Conserved hypothetical protein
PFL_4674[d]	PFL_4674	5396969..5398936	655	73.493	GGTG	None detected	Hypothetical protein PA2G_05487 from *Pseudomonas aeruginosa* 2192 (EAZ62061), 0.0	Conserved hypothetical protein
PFL_4675	*pilL*	5399717..5400862	381	40.331	AAAGG	PilL protein from plasmid ColIb-P9 (ZP_02795682), 4e-09	Type IV pilus protein PSPA7_4470 from *Pseudomonas aeruginosa* PA7 (ABR86409), 2e-103	Putative pilus biogenesis protein
PFL_4676	*pilN*	5400859..5402700	613	64.269	GAGAGA	Putative pilus formation outer membrane protein from plasmid ColIb-P9 (ZP_02783381), 7e-58	Type IV B pilus protein from *Pseudomonas syringae* pv. *syringae* B728a (AAY36567), 6e-168	Putative pilus biogenesis protein of secretin superfamily
PFL_4677	*pilO*	5402711..5404045	444	48.788	AAGGG	Hypothetical protein R64_p127 from *Salmonella typhimurium* plasmid R64 (BAA77975), 6e-10	Type IV B pilus protein from *Pseudomonas syringae* pv. *phaseolicola* (CAI36060), 3e-108	Putative pilin accessory protein
8[b]	*pilP*	5404035..5404565	176	18.384	GGAG	Pilus biogenesis protein from plasmid ColIb-P9 (NP_052544), 5e-07	Hypothetical protein from *Pseudomonas syringae* pv. *syringae* B728a (AAY36565), 8e-28	Putative pilus biogenesis protein
PFL_4678	*pilQ*	5404562..5406094	510	55.964	GGAG	Putative ATP-binding protein from plasmid ColIb-P9 (NP_052543), 2e-69	Type IV pilus protein from *Pseudomonas syringae* pv. *phaseolicola*	Putative pilus assembly; ATPase (COG2804)

							(CAI36062), 3e-160	
PFL_4679	*pilR*	5406087..5407253	388	43.386	GGAG	Putative integral membrane protein from plasmid ColIb-P9 (NP_052542), 4e-43	Type IV pilus protein from *Pseudomonas syringae* pv. *syringae* B728a (AAY36563), 2e-87	Putative type II secretory pathway component (COG1459)
PFL_4680	*pilS*	5407275..5407790	171	17.580	AGGTA	Putative type IV prepilin from plasmid ColIb-P9 (NP_052541), 1e-06	Type IV pilus protein from *Pseudomonas syringae* pv. *phaseolicola* (CAI36064), 6e-42	Putative pilus biogenesis protein
PFL_4681[d]	*pilU*	5407796..5408752	313	34.110	AGGA	None detected	Type IV pilus protein from *Pseudomonas syringae* pv. *syringae* B728a (AAY36561), 4e-86	Putative pilus retraction ATPase (COG2805)
PFL_4682	*pilV*	5408742..5409953	403	42.487	GGAG	Putative type IV prepilin from plasmid ColIb-P9 (NP_052538), 6e-42	Putative prepilin from *Pseudomonas aeruginosa* (ABR13422), 3e-91	Putative type IV prepilin
PFL_4683	*pilM*	5409968..5410408	146	15.372	AGGT	None detected	Hypothetical protein PACG_04929 from *Pseudomonas aeruginosa* C3719 (EAZ56207), 1e-28	Putative pilus biogenesis protein
9[b]	*orf9*	5411299..5411640	113	12.796	GGAG	None detected	Hypothetical protein PACG_04927 from *Pseudomonas aeruginosa* C3719 (EAZ56206), 9e-11	Conserved hypothetical protein
orf10[b]	*orf10*	5411715..5411891	58	6.147	AGGA	None detected	Hypothetical protein PACG_04926 from *Pseudomonas aeruginosa* C3719 (EAZ56204), 4e-11	Conserved hypothetical protein
11[b]	*orf11*	5411893..5412024	43	4.987	GGAG	None detected	Hypothetical protein PA14_59390 from	Conserved hypothetical

							Pseudomonas aeruginosa UCBPP-PA14 (ABJ13853), 2e-05	protein
PFL_4684	PFL_4684	5412717..5413166	149	16.192	GGAG	None detected	Hypothetical protein PSPA7_4482 from *Pseudomonas aeruginosa* PA7 (ABR85815), 8e-57	Conserved hypothetical protein
PFL_4685	PFL_4685	5414388..5414708	106	11.840	GGAG	None detected	Hypothetical protein PA14_59470 from *Pseudomonas aeruginosa* UCBPP-PA14 (ABJ13858), 9e-22	Conserved hypothetical protein
PFL_4686[cd]	PFL_4686	5414777..5415187	117	12.790	GGAG	None detected	Hypothetical protein PflO1_3006 from *Pseudomonas fluorescens* PfO-1 (ABA74744), 2e-09	Conserved hypothetical protein
PFL_4687	PFL_4687	5415235..5415528 (-)	97	10.714	GGAC	None detected	None detected	Hypothetical protein
12[b]	*orf12*	5415250..5415855	201	22.332	GGAG	None detected	Hypothetical protein PACG_04918 from *Pseudomonas aeruginosa* C3719 (EAZ56196), 1e-59	Conserved hypothetical protein
PFL_4688	PFL_4688	5415949..5416203	84	9.503	GGAG	None detected	Hypothetical protein PACG_04914 from *Pseudomonas aeruginosa* C3719 (EAZ56192), 1e-24	Hypothetical protein
13[b]	*orf13*	5416269..5416871	200	22.953	GGAGG	None detected	Hypothetical protein PaerPA_01000854 from *Pseudomonas aeruginosa* PACS2 (AAP22600), 2e-94	Conserved hypothetical protein

PFL_4689	PFL_4689	5416895..5418334	479	53.576	AGGA	None detected	Hypothetical protein from *Pseudomonas aeruginosa* (ABR13405), 0.0	Conserved hypothetical protein
PFL_4690[d]	PFL_4690	5418426..5420669	747	83.138	GGAG	None detected	Putative helicase from *Pseudomonas aeruginosa* (ABR13404), 0.0	Putative helicase (COG0553)
PFL_4691[d]	PFL_4691	5420936..5421361	141	15.326	GGAAG	RumA from IncJ plasmid R391 (AAC45831), 1e-19	Putative S24 and S26 domain peptidase from *Pseudomonas putida* W619 (ACA73238), 2e-44	Putative ultraviolet light resistance protein A (COG1974)
PFL_4692	PFL_4692	5421354..5422625	423	47.163	GCAG	RumB from IncJ plasmid R391 (AAC45832), 9e-106	RulB protein from *Pseudomonas syringae* pv. *phaseolicola* 1448A (AAZ38072), 0.0	Putative ultraviolet light resistance protein B (COG0389)
14[b]	*orf14*	5422735..5423430	231	24.539	GAGG	None detected	Hypothetical protein from *Pseudomonas aeruginosa* (ABR13380), 2e-50	Conserved hypothetical protein
PFL_4693	PFL_4693	5423444..5424187	247	27.418	AGGA	None detected	Conserved hypothetical protein PACG_04903 from *Pseudomonas aeruginosa* C3719 (EAZ56181), 5e-77	Conserved hypothetical protein
PFL_4694	PFL_4694	5424172..5424723	183	19.489	GGTG	None detected	Soluble lytic murein transglycosylase from *Pseudomonas syringae* pv. *phaseolicola* 1448A (CAI36111), 7e-162	Conserved hypothetical protein
PFL_4695	PFL_4695	5424720..5425226	168	17.681	GGTG	None detected	Hypothetical protein from *Pseudomonas aeruginosa* (ABD94658), 5e-39	Conserved hypothetical protein
PFL_4696	PFL_4696	5425475..5427691	738	82.684	GAGG	Multiple putative	TraG/TraD family	Conserved

						proteins of TraD family	protein from *Pseudomonas aeruginosa* PA7 (ABR83182), 0.0	hypothetical protein of TraG/TraD family (COG3505)
PFL_4697	PFL_4697	5427701..5428450	249	27.598	GAGG	None detected	Hypothetical protein from *Pseudomonas aeruginosa* (ABD94655), 1e-104	Conserved hypothetical protein
PFL_4698	PFL_4698	5428460..5429920	486	55.455	GAGG	None detected	Conserved hypothetical protein from *Pseudomonas aeruginosa* (ABD94654), 0.0	Putative UvrD/Rep-like helicase (COG0210)
PFL_4699	PFL_4699	5429936..5430238	100	11.450	GAGG	None detected	Conserved hypothetical protein from *Pseudomonas aeruginosa* (ABD94651), 9e-21	Conserved hypothetical protein
PFL_4700	PFL_4700	5430458..5430811	117	12.551	AGAG	Multiple putative proteins of RAQPRD family	Conserved hypothetical plasmid protein RAQPRD from *Azotobacter vinelandii* Av0P (EAM03877), 3e-34	Conserved hypothetical plasmid protein
PFL_4701	PFL_4701	5430808..5431047	79	8.151	GAGG	None detected	Hypothetical protein AvinDRAFT_0451 from *Azotobacter vinelandii* Av0P (EAM03878), 7e-19	Conserved hypothetical protein
PFL_4702	PFL_4702	5431062..5431400	112	11.728	GGAG	None detected	Hypothetical protein AvinDRAFT_0452 from *Azotobacter vinelandii* Av0P (EAM03879), 5e-31	Conserved hypothetical protein
15[b]	*orf15*	5431416..5431784	122	13.318	GAGCG	None detected	Hypothetical protein CP76 from	Conserved hypothetical

							Pseudomonas aeruginosa (AAP22568), 3e-29	protein
PFL_4703	PFL_4703	5431796..5432440	214	24.357	AGGA	None detected	Hypothetical protein AvinDRAFT_0452 from *Azotobacter vinelandii* Av0P (EAM03879), 5e-31	Conserved hypothetical protein
PFL_4704	PFL_4704	5432437..5433348	303	32.667	GGAG	None detected	Hypothetical protein AvinDRAFT_0455 from *Azotobacter vinelandii* Av0P (EAM03882), 3e-92	Conserved hypothetical protein
PFL_4705	PFL_4705	5433332..5434828	498	52.765	GGTG	None detected	Hypothetical protein PACG_04889 from *Pseudomonas aeruginosa* C3719 (EAZ56167), 0.0	Conserved hypothetical protein (COG0711)
16[b]	*orf16*	5434806..5435243	145	16.157	GGAG	None detected	Hypothetical protein PSPTO_0860 from *Pseudomonas syringae* pv. *tomato* DC3000 (AA054395), 7e-51	Conserved hypothetical protein
PFL_4706	PFL_4706	5435245..5438169	974	109.863	GAGG	None detected	Conserved hypothetical protein from *Pseudomonas aeruginosa* C3719 (EAZ56165), 0.0	Putative type IV secretory pathway component VirB4 (COG3451)
PFL_4707	PFL_4707	5438166..5438471	101	11.254	GGAG	None detected	Hypothetical protein AvinDRAFT_0459 from *Azotobacter vinelandii* Av0P (EAM03886), 2e-25	Conserved hypothetical protein
PFL_4708	PFL_4708	5438468..5439127	210	23.759	GAGG	None detected	Putative DsbA oxidoreductase from *Azotobacter vinelandii*	Putative protein disulfide

							Av0P (EAM03887), 5e-47	isomerase (COG1651)
17[b]	*orf17*	5439350..5439766	138	15.763	GAGG	None detected	Hypothetical protein PputGB1_2994 from *Pseudomonas putida* GB-1 (ABY98886), 1e-40	Putative small integral membrane protein (COG5478)
18[bc]	*orf18*	5440033..5440371	111	12.702	GAGG	None detected	Hemerythrin HHE cation binding region from *Pseudomonas fluorescens* Pf0-1 (ABA74147), 1e-25	Conserved hypothetical protein
19[b]	*orf19*	5440843..5441109	88	10.278	GGAG	None detected	Hypothetical protein PputW619_2640 from *Pseudomonas putida* W619 (ACA73132), 2e-25	Conserved hypothetical protein
PFL_4709	PFL_4709	5441688..5442116	142	15.271	GGTG	None detected	Conserved hypothetical protein PA2G_05432 from *Pseudomonas aeruginosa* 2192 (EAZ62008), 3e-29	Conserved hypothetical protein
PFL_4710	PFL_4710	5442113..5443051	312	33.486	GAGG	None detected	Hypothetical protein DUF1527 from *Azotobacter vinelandii* Av0P (EAM03889), 1e-128	Conserved hypothetical protein
PFL_4711[d]	PFL_4711	5443072..5444424	450	47.604	GGCG	None detected	Conserved hypothetical protein from *Azotobacter vinelandii* Av0P (EAM03890), 0.0	Conserved hypothetical protein
20[b]	*orf20*	5444428..5444787	119	13.561	GGAG	None detected	Conserved hypothetical protein from *Azotobacter vinelandii* Av0P (EAM03891), 5e-17	Conserved hypothetical protein
PFL_4712	PFL_4712	5444784..5446316	510	55.594	GGAG	None detected	Hypothetical protein	Conserved

							PaerPA_01000825 from *Pseudomonas aeruginosa* PACS2 (AAP22582), 0.0	hypothetical protein
21[b]	*orf21*	5446359..5446733 (-)	124	13.829	GAGA	None detected	Hypothetical protein from *Pseudomonas aeruginosa* (ABR13353), 3e-08	Conserved hypothetical protein
PFL_4713	PFL_4713	5446848..5447126	92	10.237	AGGA	None detected	Hypothetical protein PSEEN3279 from *Pseudomonas entomophila* L48 (CAK16034), 4e-33	Putative transcriptional regulator (COG3609)
22[b]	*orf22*	5447123..5447488	121	14.139	AAGCGAA	None detected	Hypothetical protein PSEEN3278 from *Pseudomonas entomophila* L48 (CAK16033), 2e-32	Putative plasmid stabilization protein ParE
23[b]	*orf23*	5448221..5448397 (-)	58	6.828	GGAGG	None detected	Conserved hypothetical protein from *Stenotrophomonas maltophilia* K279a (CAQ46011), 3e-10	Conserved hypothetical protein
PFL_4714	PFL_4714	5449040..5449906	288	32.248	GGAGG	None detected	Putative hydrolase from *Agrobacterium tumefaciens* str. C58 (AAK90870), 6e-123	Putative MhpC-like hydrolase (COG0596)
PFL_4715	PFL_4715	5449980..5451563 (-)	527	58.210	AAGGG	None detected	Putative diguanylate cyclase from *Serratia proteamaculans* 568 (ABV42169), 1e-119	Putative response regulator with CheY-like receiver and GGDEF domains (COG3706)
PFL_4716	PFL_4716	5452173..5452583	136	14.854	AAGGA	None detected	Putative response regulator receiver	Putative multi-sensor hybrid

							protein from *Pseudomonas putida* GB-1 (ABV42169), 1e-119	histidine kinase (COG2204)
24[b]	*orf24*	5452684..5452923 (-)	79	9.543	GGAAG	None detected	Hypothetical protein Pfl01_2790 from *Pseudomonas fluorescens* Pf0-1 (ABA74531), 5e-17	Conserved hypothetical protein
25[b]	*orf25*	5453369..5453590 (-)	73	7.842	GGAGG	None detected	Hypothetical protein PP_3971 from *Pseudomonas putida* KT2440 (AAN69565), 3e-15	Conserved hypothetical protein
PFL_4717	PFL_4717	5453713..5454210 (-)	165	17.874	AAGGAA	None detected	Hypothetical protein Psyr_5097 from *Pseudomonas syringae pv. syringae* B728a (AAY40124), 9e-69	Conserved hypothetical protein (COG3685)
PFL_4718	PFL_4718	5454348..5455277 (-)	309	31.963	GGAGG	None detected	Putative zinc transporter protein from *Pseudomonas fluorescens* Pf0-1 (ABA74528), 4e-131	Putative zinc transporter family protein (COG0428)
PFL_4719	PFL_4719	5455506..5456390 (-)	294	31.556	GGAG	None detected	Putative manganese containing catalase from *Pseudomonas fluorescens* Pf0-1 (ABA74529), 4e-158	Mn-containing catalase (COG3546)
PFL_4720	PFL_4720	5456952..5457452	166	17.728	GAGTA	None detected	CinA-like domain protein from *Pseudomonas fluorescens* Pf0-1 (ABA74530), 2e-65	CinA domain protein (COG1546)
26[b]	*orf26*	5457455..5457976	173	19.446	GAGG	None detected	Hypothetical protein PputGB1_3004 from *Pseudomonas putida*	Conserved hypothetical protein

							GB-1 (ABY98896), 1e-33	
PFL_4721	PFL_4721	5457984..5458430	148	16.341	GGTG	None detected	Hypothetical protein PputGB1_3005 from *Pseudomonas putida* GB-1 (ABY98897), 1e-54	Putative membrane protein (COG2323)
PFL_4722	PFL_4722	5458655..5459881 (-)	408	43.462	GAGG	None detected	Putative zinc-containing alcohol dehydrogenase from *Pseudomonas syringae pv. syringae* B728a (AAY38559), 0.0	Putative glutathione-dependent formaldehyde dehydrogenase (COG1063)
PFL_4723[c]	PFL_4723	5460380..5461593 (-)	404	44.057	AGGAA	None detected	Putative zinc-containing alcohol dehydrogenase from *Pseudomonas syringae pv. syringae* B728a (AAY38559), 0.0	Putative glutathione-dependent formaldehyde dehydrogenase (COGs1063, 1062, 1064, 0604)
27[b]	*orf27*	5461702..5461923 (-)	73	7.911	GAGG	None detected	Hypothetical protein Pput_2428 from *Pseudomonas putida* F1 (ABQ78566), 1e-16	Conserved hypothetical protein
PFL_4724[d]	PFL_4724	5462117..5462374	85	9.771	GAGAG	None detected	Hypothetical protein PSPA7_1221 from *Pseudomonas aeruginosa* PA7 (ABR81173), 2e-21	Conserved hypothetical protein
PFL_4725	PFL_4725	5462417..5463505 (-)	362	38.301	AAGGAA	None detected	Hypothetical peptidase M42 protein PputGB1_2999 from *Pseudomonas putida* GB-1 (ABY98891), 1e-124	Putative glutamyl amino peptidase (COG1363)

PFL_4726	PFL_4726	5463522..5464685 (-)	387	43.068	GAGG	None detected	Putative carboxylate-amine ligase from *Pseudomonas entomophila* L48 (CAK15852), 2e-81	Putative glutamate-cysteine ligase of 2(GCS2) family (COG2170)
PFL_4727	PFL_4727	5464698..5465678 (-)	326	35.892	GGAG	None detected	Putative methyl transferase from *Pseudomonas fluorescens* Pf0-1 (ABA73839), 8e-105	Putative methylase of HemK family (COG2890)
PFL_4728	PFL_4728	5465694..5467064 (-)	456	51.620	GGAG	None detected	Hypothetical protein from *Pseudomonas fluorescens* Pf0-1 (ABA73838), 1e-147	Conserved hypothetical protein
PFL_4729	PFL_4729	5467276..5467890	204	22.699	GGAGG	None detected	Hypothetical protein Pput_2361 from *Pseudomonas putida* F1 (ABQ78500), 1e-62	Conserved hypothetical protein
PFL_4730	PFL_4730	5467980..5468483	167	18.310	GGAG	None detected	Hypothetical protein PP_3241 from *Pseudomonas putida* KT2440 (AE016517), 6e-45	Putative outer membrane protein (COG3652)
PFL_4731	PFL_4731	5468575..5468952	125	14.628	GGAG	None detected	Putative NUDIX hydrolase Pput_2492 from *Pseudomonas putida* F1 (ABQ78627), 8e-24	Putative hydrolase of NUDIX family (COG1051)
PFL_4732	*cyoD*	5469413..5469751 (-)	112	12.422	GGAG	None detected	Putative cytochrome o ubiquinol oxidase subunit IV from *Pseudomonas putida* W619 (ACA74853), 3e-28	Putative cytochrome o ubiquinol oxidase, subunit IV (COG3125)
PFL_4733	*cyoC*	5469753..5470367 (-)	204	22.525	GGAG	None detected	Putative cytochrome o ubiquinol oxidase	Putative cytochrome o

						subunit III from *Salmonella enterica* subsp. *arizonae* (ABX22352), 1e-74	ubiquinol oxidase, subunit III (COG1845)
PFL_4734	*cyoB*	5470370..5472343 (-)	657	73.387	GGAG	None detected	Putative cytochrome-c oxidase from *Methylobacillus flagellatus* KT (ABE49562), 0.0	Putative cytochrome o ubiquinol oxidase, subunit I (COG0843)
PFL_4735	*cyoA*	5472333..5473244 (-)	303	33.283	GGAAA	None detected	Putative ubiquinol oxidase subunit II from *Ralstonia pickettii* 12J (ACD28285), 6e-90	Putative cytochrome o ubiquinol oxidase, subunit II (COG1622)
28[b]	*orf28*	5473446..5473655 (-)	69	7.364	GAGG	None detected	Putative metallothionein from *Pseudomonas putida* KT2440 (AE016519), 4e-06	Putative metallo-thionein protein
PFL_4736	PFL_4736	5474257..5474814	185	20.374	None detected	None detected	None detected	Hypothetical protein
PFL_4737	PFL_4737	5475300..5476157	285	30.055	GGAGG	None detected	Putative short-chain dehydrogenase/ reductase from *Pseudomonas fluorescens* Pf0-1 (ABA74436), 1e-132	Putative short chain dehydrogenase /reductase (COG1028)
PFL_4738	PFL_4738	5476352..5476867 (-)	171	19.338	CAGAC	None detected	Conserved hypothetical protein Pfl101_2692 from *Pseudomonas fluorescens* Pf0-1 (ABA74433), 6e-56	Conserved hypothetical protein
PFL_4739	PFL_4739	5477036..5477593	185	20.674	GGAG	None detected	Conserved hypothetical protein from *Azotobacter vinelandii*	Putative clp protease (COG0740)

							AvOP (EAM08567), 6e-37	
PFL_4740	PFL_4740	5477696..5478376	226	24.414	GGAG	None detected	Alginate lyase superfamily protein from *Pseudomonas aeruginosa* PA7 (ABR82406), 2e-69	Putative alginate lyase-like protein
PFL_4741	PFL_4741	5478387..5479724	445	48.058	GAAG	None detected	Putative sodium/hydrogen exchanger from *Pseudomonas fluorescens* Pf0-1 (ABA74434), 8e-160	Putative sodium/ hydrogen exchanger family protein (COG0025)
PFL_4742	PFL_4742	5480448..5481608	386	41.445	GGAGGA	None detected	Putative Zn-dependent alcohol dehydrogenase from *Pseudomonas stutzeri* A1501 (ABA74434), 8e-178	Putative glutathione-dependent formaldehyde dehydrogenase (COG1063)
PFL_4743	PFL_4743	5481629..5482561	310	35.571	GGGA	None detected	Hypothetical protein PST_2148 from *Pseudomonas stutzeri* A1501 (ABP79813), 3e-111	Conserved hypothetical protein (COG1801)
PFL_4744	PFL_4744	5482558..5483334	258	29.208	GAGG	None detected	Hypothetical protein PSPA7_3150 from *Pseudomonas aeruginosa* PA7 (ABR84096), 2e-94	Putative metal-dependent hydrolase (COG3568)
PFL_4745	PFL_4745	5483331..5484524	397	44.786	GGAGG	None detected	Putative phospholipase D/transphosphatidylase from *Pseudomonas putida* W619 (ACA73138), 2e-144	Putative phospholipase family protein (COG1502)
PFL_4746	PFL_4746	5484934..5485197	87	9.849	None detected	None detected	None detected	Hypothetical protein
PFL_4747	PFL_4747	5485205..5485960 (-)	251	27.823	GGTG	None detected	Hypothetical protein	Putative prolyl

							PSPA7_3136 from *Pseudomonas aeruginosa* PA7 (ABR82328), 2e-108	oligopeptidase (COG1506)
PFL_4748	PFL_4748	5485950..5487047 (-)	365	40.345	GGAGA	None detected	Hypothetical protein PA2G_01253 from *Pseudomonas aeruginosa* 2192 (EAZ58036), 5e-91	Conserved hypothetical protein
PFL_4749	PFL_4749	5487576..5487755	59	5.808	GAGG	None detected	Putative general stress protein PST_3132 from *Pseudomonas stutzeri* A1501 (ABP80761), 4e-15	Conserved hypothetical protein
PFL_4750	PFL_4750	5487797..5488909 (-)	370	40.704	GGAG	None detected	Putative sensor histidine kinase PP_3968 from *Pseudomonas putida* KT2440 (AE016590), 1e-101	Putative sensor histidine kinase (COG0642)
29[b]	*orf29*	5489044..5489640 (-)	198	21.917	GAGG	None detected	Putative DNA ligase D from *Pseudomonas putida* W619 (ACA73143), 7e-63	Putative ATP-dependent DNA ligase D
PFL_4751	PFL_4751	5489951..5491837	628	68.892	GGAG	None detected	Protein of unknown function DUF1528: relaxase from *Azotobacter vinelandii* AvOP (EAM03958), 0.0	PutativeTraI-like conjugative relaxase
PFL_4752	*int8*	5491834..5493171	445	50.922	GAGG	Multiple genome hits	Putative integrase PA2G_05424 from *Pseudomonas aeruginosa* 2192 (EAZ62001), 3e-124	Putative integrase associated with mobile island PFGI-1 (COG4973)
PFL_4753	*attR*	5493511..5493586 (-)	GGGTCGTTAGCTCAGTTGGTAGAGCAGTTGGCTTTTAACCAATTGGTCGTAGGTTCGAATCCCACAC					

(tRNA-Lys)		GACCCACCA
Alignment of attL and attR	attL	CTTTTAACCAATTGGTCGTAGGTTCGAATCCCACACGACCCACCA
		:::
	attR	CTTTTAACCAATTGGTCGTAGGTTCGAATCCCACACGACCCACCA

[a] Only expectation values of $1e^{-05}$ and below were considered as significant matches during BLAST database searches; [b] open reading frame(s) not present in the original genome annotation; [c] open reading frame contains internal frameshifts; [d] open reading frame has an alternative start site.
