# Supplementary material for: Mobile genetic elements in the genome of the beneficial rhizobacterium Pseudomonas fluorescens Pf-5
Source: BMC Microbiol. 2009 Jan 13;9:8. doi: 10.1186/1471-2180-9-8 (PMC2647930; doi:10.1186/1471-2180-9-8)
Supplement: Additional file 10 — Sequence analysis of island 02 of P. fluorescens Pf-5. Table containing annotation of mobile genetic element island 02 in the genome of Pseudomonas fluorescens Pf-5. The following information is provided for each open reading frame: locus tag number, gene name, genome coordinates, length and molecular weight of encoded protein, sequence of putative ribosome binding site, description of the closest GenBank match plus blast E-value, list of functional domains and predicted function. [file 1471-2180-9-8-S10.pdf]

Additional file 10 – Sequence analysis of island 02 of *P. fluorescens* Pf-5

CDS	Gene name	Position	Protein length (residues)	Protein mol weight (kDa)	Putative ribosome binding site	Closest protein of phage or pyocin origin (accession no.), blastp E-value [a]	Other similar proteins (accession no.), blastp E-value [a]	Predicted function (functional domains)
PFL_4976 (tRNA-Leu)	*attL*	5728474..5728491 (within tRNA-Ser – PFL_3739)	GTCTCCCTCGGGGCACCA					
PFL_4977	*int9*	5728657..5729898	413	46.144	GGAT	Site-specific recombinase from PAI-R1 island of *Pseudomonas viridiflava* ME3.1b (AAT96071), 0.0	Putative site-specific recombinase from *Acinetobacter baumannii* AYE (CAM87726), 4e-80	Putative phage integrase (COG0582)
PFL_4978	PFL_4978	5730145..5731083	312	35.389	GGTG	None detected	Hypothetical protein PAI-R1-ORF15 from *Pseudomonas viridiflava* ME3.1b (AAT96072), 3e-61	Conserved hypothetical protein
1[b]	*orf1*	5731166..5731420	84	9.114	GGTTAG	Prophage CP4-57 regulatory protein AlpA from *Azotobacter vinelandii* AvOP (EAM07670), 6e-12	Avin4386-like protein from *Pseudomonas viridiflava* ME3.1b (AAT96073), 2e-22	Putative phage regulatory protein
2[b]	*orf2*	5731417..5731722	101	11.589	GGTG	None detected	None detected	Hypothetical protein
3[b]	*orf3*	5731770..5732255	161	17.348	GAGG	None detected	None detected	Hypothetical protein
4[b]	*orf4*	5732252..5732536	94	10.009	GGTG	Hypothetical protein PAI-R1-ORF18 from *Pseudomonas viridiflava* ME3.1b (AAT96075), 2e-21	None detected	Conserved hypothetical protein
5[b]	*orf5*	5732533..5732802	89	9.801	GGTG	Hypothetical protein	Hypothetical protein	Conserved

						PAI-R1-ORF19 from *Pseudomonas viridiflava* ME3.1b (AAT96076), 4e-09	PFL_1012 from *Pseudomonas fluorescens* Pf-5 (AAY90299), 2e-13	hypothetical protein
PFL_4979[d]	PFL_4979	5732799..5735693	964	104.699	GAGG	Putative inner membrane protein PAI-R1-ORF20 from *Pseudomonas viridiflava* ME3.1b (AAT96077), 0.0	Hypothetical protein MADE_09538 from *Alteromonas macleodii* 'Deep ecotype' (EAR03332), 7e-170	Conserved hypothetical protein (COG5519)
PFL_4980	PFL_4980	5735782..5736762	326	37.070	AGGA	Hypothetical protein PAI-R1-ORF21 from *Pseudomonas viridiflava* ME3.1b (AAT96078), 3e-73	Hypothetical protein Psyr_0094 from *Pseudomonas syringae* pv. *syringae* B728a (AAY35168), 2e-52	Conserved hypothetical protein
PFL_4981	PFL_4981	5737081..5737446	121	13.823	AGGA	None detected	Putative transcriptional H-NS regulator from *Pseudomonas* sp. Y1000 (CAD98777), 4e-48	Putative transcriptional regulator/nucleoid-associated protein
6[b]	*orf6*	5738003..5738776	257	29.754	AGGA	None detected	Hypothetical protein MELB17_10963 from *Marinobacter* sp. ELB17 (EAZ97039), 2e-23	Conserved hypothetical protein
7[b]	*orf7*	5739183..5739902 (-)	239	27.510	GAGAG	None detected	None detected	Hypothetical protein
PFL_4982[d]	PFL_4982	5740075..5741337 (-)	420	46.728	GTTG	None detected	Hypothetical protein GlovDRAFT_1783 from *Geobacter lovleyi* SZ (EAV88171), 1e-43	Conserved hypothetical protein
PFL_4983	PFL_4983	5741341..5742471 (-)	376	41.241	GGAAG	None detected	Conserved hypothetical protein from *Geobacter lovleyi* SZ (EAV88150), 1e-61	Putative nucleoid-associated bacterial protein (COG3081)
PFL_4984	PFL_4984	5743437..5745014 (-)	525	60.166	AGGA	None detected	Hypothetical protein Acid345_3761 from	Putative nuclease of RecB family

			Acidobacteria bacterium Ellin345 (ABF42761), 4e-45	(COG1637)
attR	5745229..5745246	GTCTCCCTCGGGGCACCA		

```
Alignment of      attL GTCTCCCTCGGGGCACCA
attL and attR:         ::::::::::::::::::
                  attR GTCTCCCTCGGGGCACCA
```

[a] Only expectation values of $1e^{-05}$ and below were considered as significant matches during BLAST database searches; [b] open reading frame(s) not present in the original genome annotation; [c] open reading frame contains internal frameshifts; [d] open reading frame has an alternative start site.
